# Sleep patterns, genetic susceptibility, and digestive diseases: a large-scale longitudinal cohort study

**DOI:** 10.1097/JS9.0000000000001695

**Published:** 2024-05-23

**Authors:** Yuying Ma, Shiyi Yu, Qinming Li, Haifeng Zhang, Ruijie Zeng, Ruibang Luo, Qizhou Lian, Felix W. Leung, Chongyang Duan, Weihong Sha, Hao Chen

**Affiliations:** a Department of Gastroenterology, Guangdong Provincial People’s Hospital (Guangdong Academy of Medical Sciences), Southern Medical University; b The Second School of Clinical Medicine, Southern Medical University; c Department of Biostatistics, School of Public Health, Southern Medical University; d Department of Cardiology, Sun Yat-Sen Memorial Hospital, Sun Yat-Sen University; e Cord Blood Bank, Guangzhou Institute of Eugenics and Perinatology, Guangzhou Women and Children’s Medical Center, Guangzhou Medical University, Guangzhou; f HKUMed Laboratory of Cellular Therapeutics, The University of Hong Kong; g Department of Computer Science, The University of Hong Kong; h State Key Laboratory of Pharmaceutical Biotechnology, The University of Hong Kong, Hong Kong SAR; i Faculty of Synthetic Biology, Shenzhen Institute of Advanced Technology, Chinese Academy of Sciences, Shenzhen; j Shantou University Medical College, Shantou, Guangdong, China; k David Geffen School of Medicine, University of California Los Angeles, Los Angeles; l Sepulveda Ambulatory Care Center, Veterans Affairs Greater Los Angeles Healthcare System, North Hills, CA, USA

**Keywords:** cohort study, gastroenterology, genetic risk, sleep

## Abstract

**Background:**

Sleep problems are prevalent. However, the impact of sleep patterns on digestive diseases remains uncertain. Moreover, the interaction between sleep patterns and genetic predisposition with digestive diseases has not been comprehensively explored.

**Methods:**

Four hundred ten thousand five hundred eighty-six participants from UK Biobank with complete sleep information were included in the analysis. Sleep patterns were measured by sleep scores as the primary exposure, based on five healthy sleep behaviors. Individual sleep behaviors were secondary exposures. Genetic risk of the digestive diseases was characterized by polygenic risk score. Primary outcome was incidence of 16 digestive diseases.

**Results:**

Healthy sleep scores showed dose-response associations with reduced risks of digestive diseases. Compared to participants scoring 0–1, those scoring 5 showed a 28% reduced risk of any digestive disease, including a 50% decrease in irritable bowel syndrome, 37% in non-alcoholic fatty liver disease, 35% in peptic ulcer, 34% in dyspepsia, 32% in gastroesophageal reflux disease, 28% in constipation, 25% in diverticulosis, 24% in severe liver disease, and 18% in gallbladder disease, whereas no correlation was observed with inflammatory bowel disease and pancreatic disease. Participants with poor sleep and high genetic risk exhibited approximately a 60% increase in the risk of digestive diseases. A healthy sleep pattern is linked to lower digestive disease risk in participants of all genetic risk levels.

**Conclusions:**

In this large population-based cohort, a healthy sleep pattern was associated with a reduced risk of digestive diseases, regardless of genetic susceptibility. The authors’ findings underscore the potential impact of healthy sleep traits in mitigating the risk of digestive diseases.

## Introduction

Highlights

- By leveraging a large-scale cohort, we newly introduced the healthy sleep score by considering the combined impact of five sleep behaviors on the susceptibility of digestive diseases, thereby providing a more comprehensive sleep pattern.
- Our large-scale prospective study provides a robust foundation for investigating the relationship between individual sleep behaviors and digestive diseases. This substantiates and contributes further to the growing body of evidence that optimal sleep duration and being free of insomnia were associated with reduced risks across a wide range of digestive outcomes.
- We first assess the joint associations of sleep patterns and genetic risk with risks of digestive diseases. Our results suggested that participants with poor sleep and high genetic risk exhibited approximately a 60% increase in the risk of digestive diseases. A healthy sleep pattern is linked to lower digestive disease risk in participants of all genetic risk levels

Sleep problems are widespread in the general population. A recent meta-analysis, in which more than a million people across the United States, Netherlands, and United Kingdom were involved, showed ~13.3% of the adult participants suffered from poor-quality sleep^1^. Meanwhile, sleep problems are common among children and the elderly, with a prevalence rate up to 30%^2,3^. Digestive diseases represent a formidable public health challenge, significantly affecting the well-being of populations worldwide^4,5^. Surgery serves as a critical therapeutic approach for digestive system diseases. As indicated by statistics from a Global Burden of Disease Study, the surgical demand for digestive system diseases accounts for approximately 6.6% of the surgical demand for non-communicable diseases^6^. Emerging evidence has linked sleep traits with certain digestive diseases^7^. However, previous studies have led to inconsistent conclusions. For example a recent cohort study revealed that short sleep duration and napping were associated with inflammatory bowel disease (IBD)^8^, while another Mendelian randomization analysis did not support the causality between sleep characteristics and IBD^9^. Moreover, sleep traits are commonly interconnected and may collectively influence one another. Prior studies were limited as most research has primarily focused on examining the associations between individual sleep traits (e.g. sleep duration, insomnia, daytime napping) and specific digestive diseases, disregarding the comprehensive impact of an individual’s overall sleep quality. To the best of our knowledge, the existing literature remains scarce in investigating the overarching influence of sleep quality on the broader spectrum of digestive diseases.

Furthermore, previous research has demonstrated that genetic predisposition may interact with lifestyle behaviors in the development of health outcomes^10–13^. Nonetheless, the impact of a healthy sleep pattern, which encompasses a set of sleep traits, on modifying the effect of genetic susceptibility on digestive diseases remains largely unexplored.

In this study using a large prospective cohort from the UK Biobank, we aim to explore the association between the healthy sleep pattern, based on a combination of five pivotal sleep traits (sleep duration, insomnia, snoring, daytime sleepiness, and chronotype) with the risk of digestive diseases. This assessment approach serves to mitigate potential biases inherent in the selection of isolated sleep parameters. Additionally, we also investigate the association between each of the individual sleep traits and digestive diseases. Furthermore, we explore the interaction between sleep patterns and genetic predisposition with digestive diseases and the potential gene–sleep interactions.

## Methods

### Study population

**Figure 1:**
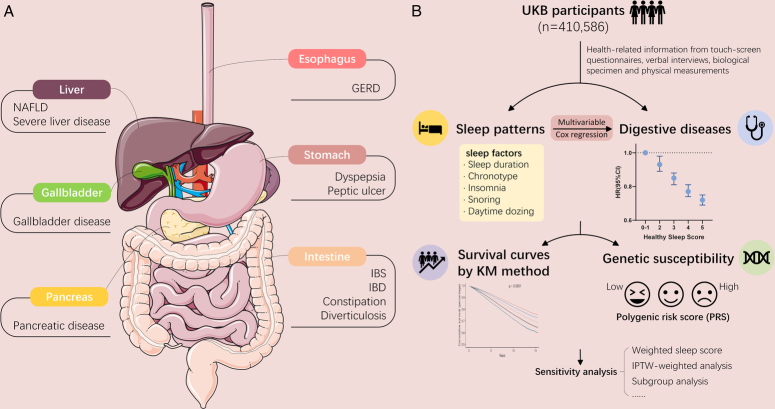
Overview of study design. (A) The main digestive system organs that suffer from digestive system diseases. Secondary outcomes are listed below each organ. (B) A healthy sleep score was constructed according to five sleep traits and defined the low-risk groups as follows: early chronotype, sleep 7–8 h per day, never/rarely insomnia, no snoring, and no frequent excessive daytime sleepiness. Digestive diseases were defined based on ICD-10. Cox proportional hazards models were used to investigate the association between sleep patterns and the risk of digestive diseases. Genetic risk was characterized by polygenic risk score. UKB, UK Biobank. ICD-10, the 10th revision of the International Classification of Diseases. Image was cited from smart.servier.com. GERD: gastroesophageal reflux disease; IBD, inflammatory bowel disease; IBS, irritable bowel syndrome; KM, Kaplan–Meier; NAFLD, non-alcohol fatty liver disease.

The UK Biobank is an ongoing large-scale prospective study with more than 500 000 participants aged from 37 to 73 recruited in 2006–2010. It provided information about sleep and various health-related aspects, which have been gathered through baseline or follow-up touch-screen questionnaires, verbal interviews, biological specimens and physical measurements. Subsequently, three repeated assessments were conducted with a decreased enrollment of participants. Hospital inpatient data were consistently updated through connections with the Hospital Episode Statistics for England, Scottish Morbidity Record for Scotland, and Patient Episode Database for Wales. Mortality data are acquired through linkages to National Health Service (NHS) Digital and NHS Central Register. The UK Biobank research has obtained approval from the North West Multi-Centre Research Ethics Committee and all enrolled participants provided their written informed consent. Further details about the UK Biobank can be found elsewhere^14^. Figure 1 demonstrates the study design.

**Figure 2:**
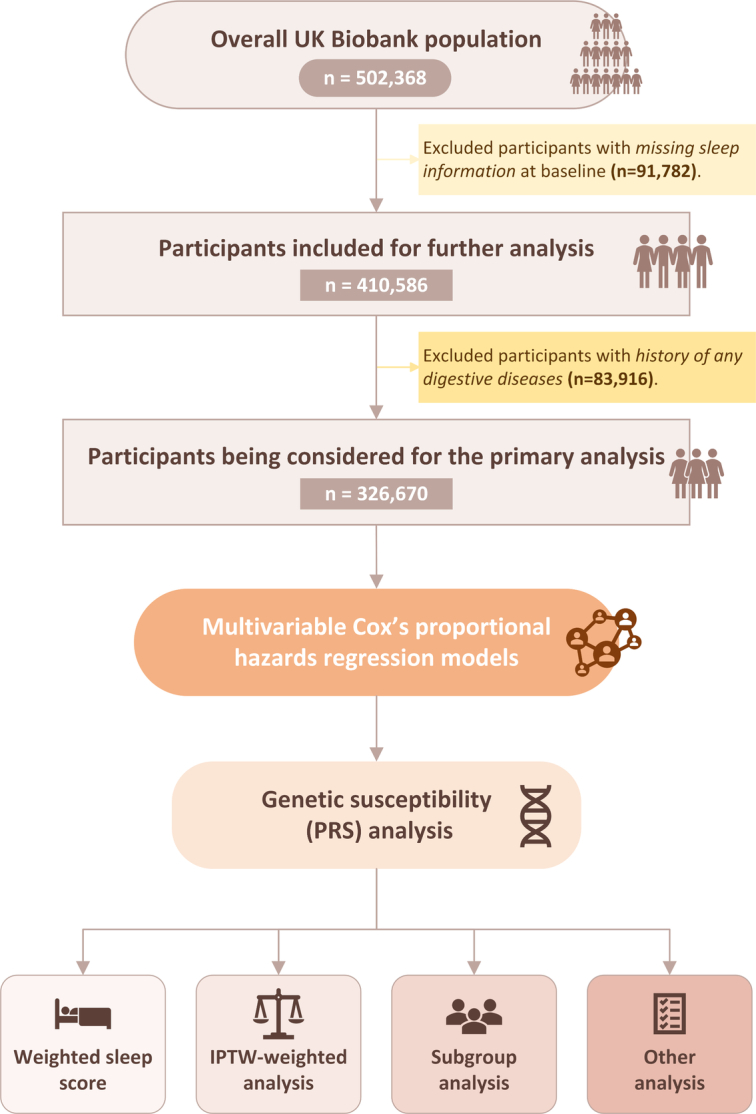
Flow chart of eligible participants’ selection. IPTW, inverse probability of treatment weighting; PRS, ploygenic risk score.

In this longitudinal cohort study, participants with missing information on sleep (*n*=91 782) were excluded at baseline, resulting in 410 586 participants being considered for further analysis. To assess selection bias, we compared baseline characteristics between excluded and included populations. For the primary outcome (a composite of digestive diseases) analysis, individuals with history of any digestive diseases (*n*=83 916) were excluded, leaving 326 670 participants in total (Fig. 2).

### Assessment of sleep traits

The data of sleep trait is obtained from the electronic touchscreen questionnaire designed by the UK Biobank at baseline. To assess the consistency of sleep information over time, we determined the number of individuals whose sleep information at each follow-up point remained consistent with their baseline data. Then we calculated the proportion of individuals whose sleep information remained consistent with their baseline data, expressed as a percentage of the total number of participants included in each follow-up assessment. This data exhibited substantial consistency with subsequent repeated assessments conducted between 2014 and 2023, demonstrating consistent rates ranging from 70 to 97% (Supplementary Table 1, Supplemental Digital Content 2, http://links.lww.com/JS9/C654). We included five sleep-related evaluative parameters covering chronotype, sleep duration, snoring, sleeplessness and daytime dozing, all of which are self-reported. The specific criteria for evaluating sleep trait are listed in Supplementary Table 2, Supplemental Digital Content 2, http://links.lww.com/JS9/C654.

### Definition of a healthy sleep score and sleep patterns

The constitution of a healthy sleep score encompasses a combination of the five distinct sleep traits. This particular aggregation of sleep-related factors has been employed in previous research^15^. In the present study, we used it as the primary exposure of interest, with secondary exposure being performed on each individual sleep-related factor. The categorization of questionnaire-derived sleep-related factors is defined as follows:(1) factors regarded as a healthy sleep pattern were: “morning chronotype”, “sleep for 7-8 hours per day^16^”, “Never or rarely experience insomnia symptoms”, “I don’t often feel sleepy during the day”, “No self-reported snoring”. (2) Participants categorized within the low-risk spectrum were assigned a score of 1 point, while those classified under high-risk a score of 0 points. (3) The cumulative total of these five individual scores generates the composite healthy sleep score, which spans a spectrum from 0 to 5. A higher resultant score is indicative of a healthier pattern of sleep. (4) Utilizing the derived sleep score, the sleep pattern is categorized into three groups: “poor sleep” (sleep score ≤ 1), “intermediate sleep” (2 ≤ sleep score ≤ 3), “healthy sleep” (sleep score ≥ 4).

In sensitivity analysis, we advanced our study by developing a weighted sleep score. This score was formulated through the incorporation of five distinct sleep parameters, utilizing the following mathematical expression: weighted sleep score=(β1×factor1+β2×factor2+…β5×factor5) × (5/sum of the β coefficients). Subsequent to the process of linear normalization, the weighted sleep score was transformed to a spectrum ranging between 0 and 5. This transformation takes into account the magnitudes of the adjusted relative risks associated with each parameter, thereby providing a representation that encapsulates the cumulative effect of the five parameters on sleep patterns.

### Definition of genetic risk

Details of polygenic risk scores (PRS) calculation can be found in Supplementary materials, Supplemental Digital Content 2, http://links.lww.com/JS9/C654. Different levels of genetic susceptibility of participants were determined as low (quintile 1), intermediate (quintile 2–4), or high (quintile 5) or genetic susceptibility based on the five quantiles defined by the PRS for each outcome of digestive diseases.

### Assessment of outcomes

The health outcomes of patients in UK Biobank are mainly obtained through linkage to electronic medical and updated on a regular basis. In our study, the primary outcome was a composite of digestive diseases, encompassed by the secondary outcome defined in accordance with the tenth edition of the International Classification of Diseases (ICD-10) based on evidence in prior literature^8,9,17–20^: dyspepsia, peptic ulcer (gastric ulcers, duodenal ulcers, other peptic ulcer), irritable bowel syndrome (IBS), constipation, gastroesophageal reflux disease (GERD), severe liver disease, inflammatory bowel disease (IBD) (Crohn’s disease and ulcerative colitis), gallbladder disease (cholelithiasis and cholecystitis), non-alcoholic fatty liver disease (NAFLD), pancreatic diseases (chronic pancreatitis, acute pancreatitis, pancreatic cyst, other pancreatic diseases), and diverticulosis. Details of the ICD-10 codes can be found in Supplementary Table 3, Supplemental Digital Content 2, http://links.lww.com/JS9/C654.

### Covariates

Covariates were obtained from baseline data, which were extracted from touchscreen questionnaire, verbal interview, physical measurements, health-related records, *etc.* The selection of specific covariates for analysis was guided by a review of extant literature^13-18^ and an investigative evaluation of directed acyclic graphs (DAGs), as illustrated in Supplementary Fig 1, Supplemental Digital Content 2, http://links.lww.com/JS9/C654. The array of covariates incorporated into the study encompasses sociodemographic factors, including age, sex, ethnicity, household income, the Townsend deprivation index (TDI), and educational attainment; lifestyle attributes such as smoking habits, alcohol intake, and physical activity levels; BMI; utilization of acid inhibitors; the number of hospital admissions within the three years leading up to the index date (proxy of healthcare utilization), serving as a surrogate indicator of healthcare utilization; and a range of existing comorbid conditions, including anxiety, depression, hypertension, heart failure, renal failure, diabetes, thyroid diseases, dementia, myocardial infarction (MI), asthma, chronic obstructive pulmonary disease (COPD), and stroke.

In addressing the issue of missing data within the covariates, we quantified both the number and percentage of missing values, employing the chain equation methodology (utilizing the MICE package in R^21^) to facilitate multiple imputation and predictive mean matching techniques. This method combines regression models and nearest-neighbor matching to handle missing data. Five datasets were imputed with 50 iterations each.

### Statistical analysis

The participants’ baseline characteristics were described using means or percentages according to the distribution of healthy sleep score. The follow-up duration was defined as the time interval between the baseline date and the date of the first diagnosis of outcomes, death, or the censoring date (30 October 2022), whichever occurred first. To assess the occurrence of digestive diseases, the Cox proportional hazards model was applied to estimate the hazard ratios (HRs) and 95% CIs.

Accounting for various confounding factors, comprehensive adjustments were implemented in different models. In Model 1, age and sex were adjusted. Additionally, Model 2 incorporated adjustments for ethnicity, BMI, TDI, household income, education, physical activity, acid inhibitor use, recent hospital admissions, smoking status and alcohol consumption. Model 3 extended the adjustments in Model 2 by including comorbidities (hypertension, heart failure, MI, stroke, asthma, renal failure, COPD, thyroid disease, anxiety, depression, dementia, and diabetes). Then, Kaplan–Meier (KM) curve was applied to visualize the survival data of participants. In the secondary analysis of each sleep trait, we divided each sleep trait into low or high (reference) risk groups.

Further exploration of the interaction between healthy sleep score and genetic susceptibility to digestive diseases was conducted based on post hoc analysis for statistically significant digestive system outcomes identified in the primary analysis. To test for interaction between healthy sleep score and genetic predisposition of each digestive outcome, we examined statistical interaction by incorporating the two variables and their cross-product term in the Model 3, respectively. Likelihood ratio test was used to compare models with and without a cross-product term.

To validate the robustness of our results, a series of sensitivity analyses were conducted. Firstly, inverse probability weights were computed for each participant to minimize confounders^22^. To verify the validity of weighting, we assessed the standardized mean difference (SMD) among covariates within the weighted populations. A SMD less than 0.1 was regarded as evidence of adequate balance of covariates between groups. Cox regression models were then developed using inverse probability weights. Secondly, we excluded participants diagnosed with digestive diseases within 2 years after baseline to reduce the risk of reverse causality. Thirdly, we limited the censoring date to 31 December 2019, to factor in the onset of the COVID-19 pandemic. Fourthly, we excluded individuals who reported a sleep duration of less than 4 h or exceeding 11 hours. Lastly, we excluded individuals with missing covariate information. Additionally, we conducted subgroup analysis and tested the potential interactions between sleep patterns and age, gender, ethnicity, BMI, the TDI, physical activity, household income, smoking status, alcohol consumption, acid inhibitor use, recent hospital admissions, education, comorbidities by adding multiplication interaction terms between healthy sleep scores and potential modifiers to the model.

The work has been reported in line with the STROCSS criteria^23^, Supplemental Digital Content 1, http://links.lww.com/JS9/C653. Statistical analyses were conducted utilizing R 4.2.1 software. Two-sided statistical tests were employed, and significance was defined as *P* < 0.05.

### Patient and Public Involvement

Patients or the public were not involved in the design, or conduct, or reporting, or dissemination plans of our research.

## Results

### Baseline characteristics

The median (interquartile range) follow-up time was 13.2 (11.4–14.1) years. The baseline characteristics of the study population based on the healthy sleep score are shown in Table 1. Supplementary Table 4, Supplemental Digital Content 2, http://links.lww.com/JS9/C654 showed baseline comparisons between excluded and included populations, where no significant difference was found. The mean age of 410 586 participants was 56.5 years (standard deviation: 8.09), with females accounting for 225 923 (55.0%). The distribution of sleep scores at baseline assessment was as follows: 0–1 (2.5%), 2 (11.3%), 3 (28.1%), 4 (36.7%), and 5 (21.4%), respectively. Notably, participants with higher healthy sleep scores exhibited a greater likelihood of engaging in physical activity and nonsmokers, as well as having lower BMI, TDI and a lower prevalence of comorbidities including anxiety, depression, hypertension, heart failure, renal failure, asthma, COPD, diabetes and thyroid disease (Table 1). Details on the number and proportion of missing covariates can be found in Supplementary Table 5, Supplemental Digital Content 2, http://links.lww.com/JS9/C654.

**Table 1:** Baseline characteristics of the study population.

	Healthy sleep score					
Baseline characteristics	0–1	2	3	4	5	Overall
No. participants, *n* (%)	9984 (2.5)	46 416 (11.3)	115 572 (28.1)	150 875 (36.7)	87 739 (21.4)	410 586
Sex, female, *n* (%)	5100 (51.1)	24 394 (52.6)	59 721 (51.7)	81 448 (54.0)	55 260 (63.0)	225 923 (55.0)
Age, mean (SD), years	56.6 (7.73)	56.7 (7.83)	56.7 (7.97)	56.4 (8.15)	56.2 (8.31)	56.5 (8.09)
Ethnicity, White, *n* (%)	9291 (93.1)	43 909 (94.6)	109 726 (94.9)	143 524 (95.1)	4058 (95.4)	390 131 (95.0)
BMI, mean (SD), kg/m^2^	30.1 (5.87)	28.7 (5.27)	27.9 (4.83)	27.1 (4.54)	26.2 (4.27)	27.4 (4.77)
Deprivation index, mean (SD)	−0.584 (3.38)	−1.04 (3.21)	−1.32 (3.08)	−1.51 (2.97)	−1.62 (2.91)	−1.41 (3.03)
Physical activity, mean (SD), MET min/week	2360 (2780)	2470 (2710)	2590 (2710)	2680 (2690)	2820 (2700)	2650 (2700)
Household income						
<18 000, *n* (%)	3122 (31.3)	11 894 (25.6)	25 537 (22.1)	29 595 (19.6)	16 730 (19.1)	86 878 (21.2)
18 000–30 999, *n* (%)	2555 (25.6)	12 128 (26.1)	29 805 (25.8)	37 967 (25.2)	21 859 (24.9)	104 314 (25.4)
31 000–51 999, n (%)	2337 (23.4)	11 694 (25.2)	30 404 (26.3)	40 626 (26.9)	23 443 (26.7)	108 504 (26.4)
52 000–100 000, *n* (%)	1616 (16.2)	8556 (18.4)	23 486 (20.3)	33 308 (22.1)	19 843 (22.6)	86 809 (21.1)
>100 000, *n* (%)	354 (3.5)	2144 (4.6)	6340 (5.5)	9379 (6.2)	5864 (6.7)	24 081 (5.9)
Alcohol consumption						
Daily or almost daily, *n* (%)	2049 (20.5)	10 136 (21.8)	25 273 (21.9)	31 672 (21.0)	15 884 (18.1)	85 014 (20.7)
Three or four times a week, *n* (%)	1895 (19.0)	9918 (21.4)	27 025 (23.4)	36 589 (24.3)	20 986 (23.9)	96 413 (23.5)
Once or twice a week, *n* (%)	2385 (23.9)	11 562 (24.9)	29 355 (25.4)	39 654 (26.3)	23 810 (27.1)	106 766 (26.0)
One to three times a month, *n* (%)	1188 (11.9)	5198 (11.2)	12 574 (10.9)	16 182 (10.7)	10 116 (11.5)	45 258 (11.0)
Special occasions only or never, *n* (%)	1451 (14.5)	5789 (12.5)	12 702 (11.0)	15 816 (10.5)	9904 (11.3)	45 662 (11.1)
Never, *n* (%)	1016 (10.2)	3813 (8.2)	8643 (7.5)	10 962 (7.3)	7039 (8.0)	31 473 (7.7)
Smoking status						
Never smoker, *n* (%)	4272 (42.8)	21 678 (46.7)	59 176 (51.2)	84 114 (55.8)	54 542 (62.2)	223 782 (54.5)
Previous smoker, *n* (%)	3914 (39.2)	17 869 (38.5)	42 462 (36.7)	52 480 (34.8)	27 294 (31.1)	144 019 (35.1)
Current smoker, *n* (%)	1798 (18.0)	6869 (14.8)	13 934 (12.1)	14 281 (9.5)	5903 (6.7)	42 785 (10.4)
Acid inhibitor use, *n* (%)	466 (4.7)	1934 (4.2)	4134 (3.6)	4614 (3.1)	2212 (2.5)	13 360 (3.3)
Recent hospital admissions, Mean (SD)	0.883 (1.50)	0.693 (1.28)	0.569 (1.12)	0.481 (1.00)	0.436 (0.941)	0.530 (1.08)
Comorbidities						
Anxiety, *n* (%)	662 (6.6)	2407 (5.2)	4875 (4.2)	4956 (3.3)	2536 (2.9)	15 436 (3.8)
Depression, *n* (%)	1812 (18.1)	6116 (13.2)	10 665 (9.2)	10 123 (6.7)	4798 (5.5)	33 514 (8.2)
Hypertension, *n* (%)	4005 (40.1)	15 176 (32.7)	33 531 (29.0)	37 636 (24.9)	18 643 (21.2)	108 991 (26.5)
Heart failure, *n* (%)	114 (1.1)	350 (0.8)	660 (0.6)	721 (0.5)	309 (0.4)	2154 (0.5)
Renal failure, *n* (%)	206 (2.1)	715 (1.5)	1475 (1.3)	1628 (1.1)	895 (1.0)	4919 (1.2)
Asthma, *n* (%)	1793 (18.0)	6752 (14.5)	14 463 (12.5)	16 858 (11.2)	9040 (10.3)	48 906 (11.9)
COPD, *n* (%)	505 (5.1)	1498 (3.2)	2395 (2.1)	2295 (1.5)	961 (1.1)	7654 (1.9)
Diabetes, *n* (%)	4143 (41.5)	15 724 (33.9)	34 578 (29.9)	38 826 (25.7)	19 154 (21.8)	112 425 (27.4)
Thyroid disease, *n* (%)	898 (9.0)	3446 (7.4)	7873 (6.8)	9432 (6.3)	5583 (6.4)	27 232 (6.6)

COPD, chronic obstructive pulmonary disease; MET, metabolic equivalent of task.

### Association of healthy sleep score with digestive diseases

**Figure 3:**
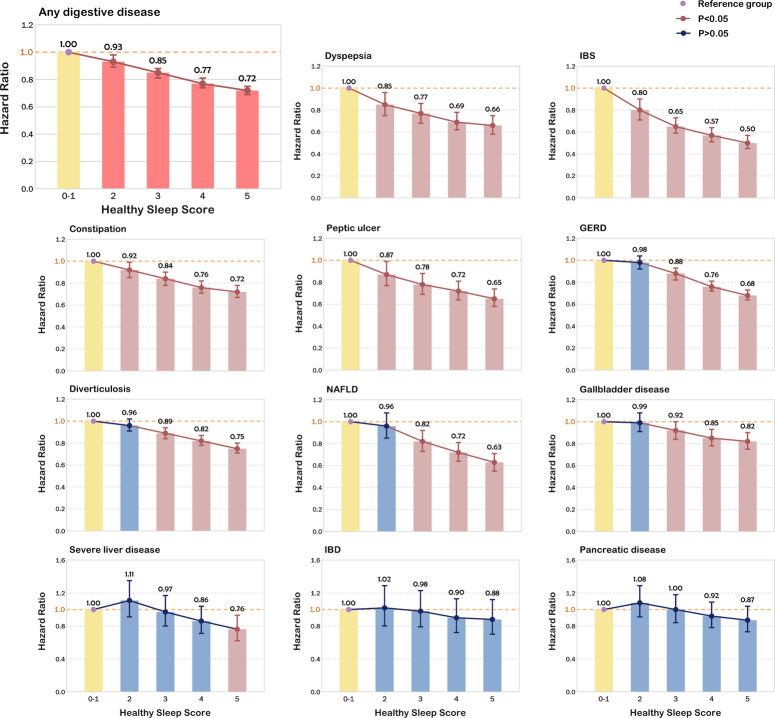
Association of healthy sleep score with digestive disease. Incident risk of digestive diseases according to healthy sleep score. Incident risk of digestive diseases according to healthy sleep score in model adjusted for age, sex, ethnicity, BMI, Townsend Deprivation Index, household income, education, acid inhibitor use, recent hospital admissions, smoking status, alcohol consumption and physical activity and comorbidities, including hypertension, heart failure, myocardial infarction, stroke, asthma, renal failure, chronic obstructive pulmonary disease, thyroid disease, anxiety, depression, dementia, and diabetes. GERD, gastroesophageal reflux disease; IBD, inflammatory bowel disease; IBS, irritable bowel syndrome; NAFLD, non-alcoholic fatty liver disease.

When all five sleep-related factors were incorporated into a healthy sleep score, we observed dose-response associations between the increase in the healthy sleep score and the decrease in the risk of digestive diseases (Fig. 3 and Supplementary Table 6, Supplemental Digital Content 2, http://links.lww.com/JS9/C654). Compared with participants who scored 0 to 1, the HRs (95% CI) were 0.93 (0.89–0.98) for those scoring 2, 0.85 (0.81–0.88) for those scoring 3, 0.77 (0.74–0.81) for those scoring 4, 0.72 (0.69–0.75) for those scoring 5. Participants with a healthy sleep score of 5 had the lowest risk for digestive diseases, and the HRs (95% CI) was 0.72 (0.69–0.75) for any digestive disease, 0.66 (0.58–0.75) for dyspepsia, 0.50 (0.45–0.57) for IBS, 0.72 (0.67–0.78) for constipation, 0.65 (0.58–0.74) for peptic ulcer, 0.68 (0.64–0.73) for GERD, 0.82 (0.75–0.90) for gallbladder disease, 0.76 (0.62–0.93) for severe liver disease, 0.63 (0.55–0.71) for NAFLD and 0.75 (0.71–0.80) for diverticulosis. All these associations were statistically significant (all *P*<0.01). Our analysis also indicated no statistically significant association between healthy sleep score and IBD (HR 0.88; 95% CI 0.70–1.12; *P*=0.30) or pancreatic disease (HR 0.87; 95% CI 0.73–1.04; *P*=0.13). Supplementary Figure 2, Supplemental Digital Content 2, http://links.lww.com/JS9/C654 showed the KM curve to visualize the survival data.

### Association of five sleep traits with incident digestive diseases

Supplementary Table 7, Supplemental Digital Content 2, http://links.lww.com/JS9/C654 presented the associations between individual sleep traits and digestive outcomes. Compared with high-risk groups of the corresponding sleep trait, never/rarely insomnia, early chronotype, daily sleep duration 7–8 h, and no frequent daytime sleepiness were each independently associated with any digestive diseases after full adjustment, with a 16% (14–17%), 6% (4–7%), 15% (12–18%), 10% (8–11%) and 5% (4–6%) lower risk, respectively. Additionally, daily sleep duration of 7–8 h was associated with all subtypes: dyspepsia (HR 0.88, 95 CI% 0.84–0.92), IBS (HR 0.82, 95 CI% 0.78–0.86), constipation (HR 0.88, 95 CI% 0.85–0.90), peptic ulcer (0.85, 95 CI% 0.82–0.90), GERD (HR 0.88, 95 CI% 0.86–0.90), IBD (HR 0.84, 95 CI% 0.78–0.91), gallbladder disease (HR 0.96, 0.93–0.99), sever liver disease (HR 0.77, 95 CI% 0.71–0.83), NAFLD (HR 0.86, 95 CI% 0.81–0.90), pancreatic disease (HR 0.92, 95 CI% 0.86–0.98), and diverticulosis (HR 0.93, 95 CI% 0.91–0.95). Free of insomnia was also associated with a decreased risk of developing a range of digestive diseases, except for IBD; early chronotype is associated with a lower risk of various digestive diseases, excluding IBD and pancreatic disease; no frequent daytime sleepiness is also associated with lower risk of most of the digestive diseases, excluding IBD, gallbladder disease, severe liver disease and pancreatic disease; while no snoring was only associated with decreased risks of GERD, gallbladder disease, NAFLD and diverticulosis.

Overall, when all five sleep traits were classified as low or high (reference) risk groups, participants with low-risk sleep traits showed decreased risks of any digestive diseases (HR 0.87, 95% CI 0.86–0.89) or other subtypes.

### Interaction between sleep pattern and genetic risk with digestive diseases

**Figure 4:**
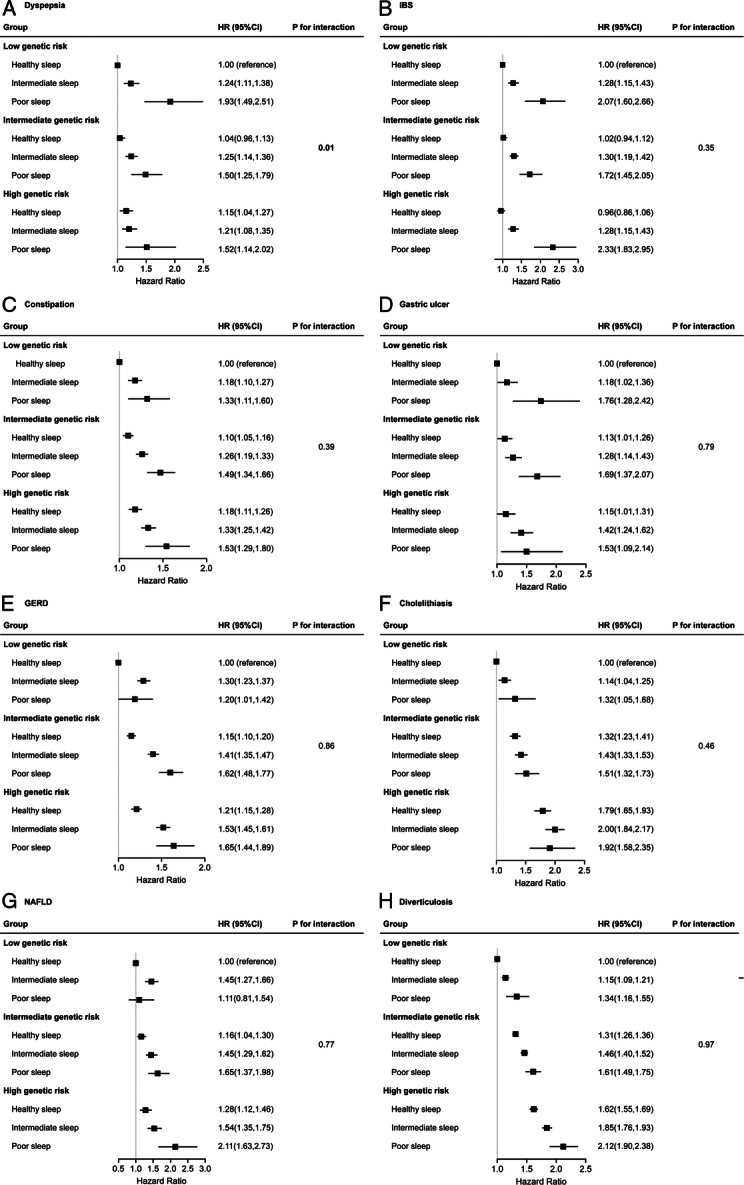
The interaction of genetic risk and sleep pattern with digestive diseases. (A) Dyspepsia; (B) IBS; (C) Constipation; (D) Gastric ulcer; (E) GERD; (F) Cholelithiasis; (G) NAFLD; (H) Diverticulosis. Multivariable model was adjusted for age, sex, ethnicity, BMI, the Townsend Deprivation Index, physical activity, household income, smoking status, alcohol consumption, acid inhibitor use, recent hospital admissions, education, and comorbidities. The vertical line indicates the reference value of 1. GERD, gastroesophageal reflux disease; HR, hazard ratio; IBD, inflammatory bowel disease; IBS, irritable bowel syndrome; NAFLD, non-alcoholic fatty liver disease.

Figure 4 shows the combined correlation between sleep pattern, genetic susceptibility and dyspepsia, IBS, constipation, gastric ulcer, GERD, cholelithiasis, NAFLD, and diverticulosis. In this joint analysis, a healthy sleep pattern reduced the risk of digestive outcomes in populations with low, intermediate, high genetic risk, whereas no interaction was observed between sleep patterns and genetic susceptibility for most digestive outcomes, except for dyspepsia (P for interaction=0.01). Compared to participants with healthy sleep patterns in low genetic risk group, those with poor sleep patterns in high genetic risk group had a significant increase in risks of digestive outcomes: dyspepsia (HR 1.52, 95% CI 1.14–2.02), IBS (HR 2.33, 95% CI 1.83–2.95), constipation (HR 1.53, 95% CI 1.29–1.80), gastric ulcer (HR 1.53, 95% CI 2.14), GERD (HR 1.65, 95% CI 1.44–1.89), cholelithiasis (HR 1.92, 95% CI 1.58–2.35), NAFLD (HR 2.11, 95% CI 1.63–2.73), diverticulosis (HR 2.12, 95% CI 1.90–2.38). This suggests that the protective effect of a healthy sleep pattern is statistically independent of genetic risk for digestive diseases of an individual’s level of PA.

### Sensitivity analysis

Baseline characteristics between groups after IPTW were provided in Supplementary Table 8, Supplemental Digital Content 2, http://links.lww.com/JS9/C654. All covariates exhibited an excellent balance after implementing the weighting. The results in the sensitivity analysis were generally aligned with the primary analyses after using inverse probability weights (Supplementary Table 9, Supplemental Digital Content 2, http://links.lww.com/JS9/C654), excluding individuals with less than 4 or more than 11 h of sleep (Supplementary Table 10, Supplemental Digital Content 2, http://links.lww.com/JS9/C654), participants with a diagnosis of digestive disease within 2 years after baseline (Supplementary Table 11, Supplemental Digital Content 2, http://links.lww.com/JS9/C654), participants with missing information on covariates (Supplementary Table 12, Supplemental Digital Content 2, http://links.lww.com/JS9/C654), or adjusting censoring date to the period before the COVID-19 pandemic (Supplementary Table 13, Supplemental Digital Content 2, http://links.lww.com/JS9/C654). There were no significant modifications observed in the results related to the weighted sleep score (Supplementary Table 14, Supplemental Digital Content 2, http://links.lww.com/JS9/C654). Further evaluations within subgroups were conducted to evaluate the associations between the healthy sleep score (one-point increase) and the risk of digestive diseases in each subgroup (Supplementary Table 15, Supplemental Digital Content 2, http://links.lww.com/JS9/C654). The associations between an increase of one point in healthy sleep score and each digestive disease were broadly similar among subgroups including sex, age, ethnicity, TDI, BMI, smoking status, diabetes, hypertension, and other relevant variables.

## Discussion

In this large-scale cohort study, we observed a dose-response association that higher healthy sleep scores were linked to lower risks of digestive diseases (including dyspepsia, IBS, constipation, GERD, peptic ulcer, gallbladder disease, NAFLD and diverticulosis), whereas no correlation was observed with IBD and pancreatic disease. Similar trends were observed in the analyses using weighted sleep scores, which summarizing the collective influence of the five individual sleep traits on sleep patterns by further adjusting their relative risks. For individual sleep traits, sleep duration and insomnia were associated with a wide range of digestive outcomes. Besides, participants with a poor sleep pattern faced a significant increase in the risk of digestive diseases in the high genetic risk group. Furthermore, the protective effect of healthy sleep patterns on digestive diseases was consistent across different groups of genetic risk. Our findings highlight the crucial role of healthy sleep patterns in promoting digestive system health.

Our finding is in line with prior research suggesting that a better sleep quality, which is closely related to healthy sleep patterns investigated in this study, assessed by the Pittsburgh Sleep Quality Index (PSQI) including 7 indicators of sleep^24^ was associated with reduced risks of dyspepsia^25^, GERD^26^ and NAFLD^27^. However, the assessment of PSQI did not incorporate circadian biology, a crucial mechanism with substantial implications for the regulation of gut function^28,29^. In the current study, we newly introduced the healthy sleep score by considering the joint effect of five sleep traits on the susceptibility of digestive diseases, provided a more comprehensive sleep pattern, and revealed the dose-response effect of a healthy sleep pattern on reducing risks of digestive disease in a more comprehensive approach for the first time. Considering that sleep-related factors are commonly intercorrelated, the assessment of the synergy among these sleep traits is of paramount importance. Applying the overall sleep pattern not only offers a positive framework for understanding sleep but also holds significance in advancing effective healthcare management. Additionally, implementation of a simplified rating system of sleep traits provides a comprehensible framework for the general public, incentivizing proactive efforts to enhance their sleep practices and consequently reduce the susceptibility to digestive system disorders.

For individual sleep traits, there was prior evidence that chronotype was associated with the risk of digestive diseases^30–32^, which was also confirmed in our current study. Despite prior research indicating the relationships between individual sleep traits and digestive diseases^33–36^, our study yielded critical insights for promoting healthy sleep patterns against the risks of developing digestive diseases. We found that optimal sleep duration and being free of insomnia were associated with reduced risks across a wide range of digestive outcomes. In contrast to previous studies limited by unrepresentative study populations or flawed study designs^34,37,38^, our large-scale longitudinal study provides a robust foundation for investigating the relationship between each sleep element and digestive diseases. This substantiates and contributes further to the growing body of evidence in this field. Our finding indicates the crucial roles adequate sleep duration and the absence of insomnia play in promoting digestive system health, emphasizing the need for targeted health promotion campaigns.

Furthermore, to our knowledge, our study is the first to explore the interactions between sleep patterns and genetic predisposition and digestive diseases. We found the protective effect of healthy sleep patterns on digestive diseases was consistent across different groups of genetic risk, whereas no interaction was observed between sleep patterns and genetic susceptibility for most digestive outcomes, except for dyspepsia. Participants with a poor sleep pattern and a high genetic risk group faced a significant increase in the risk of digestive diseases. Our results demonstrated that a healthy sleep pattern is an independent protective factor for digestive diseases, regardless of genetic predispositions. A high genetic predisposition may, to some extent, be mitigated by a healthy sleep pattern. Meanwhile, individuals with low genetic predisposition may cause a loss of their inherent protection if they follow poor sleep patterns. Hence, a healthy sleep pattern may assume an essential role in the primary prevention of digestive diseases across the general population, irrespective of their genetic risk profiles. This particularly provides effective guidance for high-risk populations.

Several possible mechanisms may contribute to the protective effect against healthy sleep patterns on digestive diseases. Sleep deficiency promotes the release of inflammatory cytokines^39^ and leads to chronic, systemic low-grade inflammation^40,41^ consequently elevating susceptibility to digestive system disorders^42^. Besides, poor sleep is associated with intestinal barrier dysfunction causing by intestinal mucosal injury and microbiota dysbiosis^43,44^. In particular, sleep disturbance correlates with the activation of the hypothalamic-pituitary-adrenal axis, changes in the composition of gut bacteria, and a decrease in intestinal barrier function^45^. Additionally, the gut-brain axis operates by coordinating intestinal permeability, enteroendocrine signaling, and immune activation, crucial for regulating digestion and supporting gut immunity^46^. Poor sleep could disrupt balance, potentially increasing susceptibility to gastrointestinal disorders.

The discovery of the connection between sleep patterns and the incidence of digestive diseases holds substantial implications for clinical and public health practices. Clinicians may integrate sleep quality assessments into regular check-ups, particularly for those at heightened risk of digestive ailments, and advise on lifestyle changes to mitigate these risks. By elucidating the relationship between sleep patterns and digestive system diseases, our study enhances the risk assessment framework for these conditions, potentially reducing the burden of disease and subsequent surgical interventions. Personalized sleep interventions implemented pre- and post-surgery may impact patient recovery outcomes in digestive system diseases. This insight paves the way for more tailored treatment methods, where sleep improvement becomes an integral part of managing digestive disorders alongside conventional treatments. In the public health domain, this finding could spur campaigns to elevate awareness about the role of good sleep in maintaining digestive health. Additionally, this correlation could redirect research to further explore the interplay between sleep and digestive health, potentially unveiling novel therapeutic avenues.

### Strength and limitations

There are several major strengths in our study. Firstly, the cohort’s substantial sample size ensured ample statistical power. Secondly, detailed information available in the UK Biobank provided the feasibility for adjusting for a broad range of covariates, thus minimizing the confounding bias. Thirdly, we performed a variety of sensitive analyses (e.g. IPTW, subgroup analysis etc.) to test the robustness of our results.

Despite its strength, our study has some limitations. Firstly, due to the inherent constraints of observational analysis, the causal relationship between sleep patterns and digestive diseases cannot be established. However, the observed dose-dependent association of sleep score on the risk of digestive diseases may provide evidence of causality to some extent. Secondly, the data of sleep traits was obtained from self-reported questionnaires, leading to the inevitable measurement error. However, a strong correlation had been reported between self-reported or subjective sleep traits and objective measurements^47,48^. Further studies assessing sleep quality with more objective devices (e.g. polysomnography) are needed to validate our findings. Besides, our assessments before the onset of digestive outcomes mitigates the probability of systematic biases which may influence the reported data. Thirdly, we utilized the baseline sleep trait data. Nevertheless, it demonstrated substantial agreement with the follow-up information, ranging from 70 to 97% in consistency. Fourthly, the possibility of reverse causality still exists. However, we have performed analysis to lower the risk of reverse causation bias with the exclusion of participants with a history of digestive outcomes before baseline in the main analysis or patients with a diagnosis of digestive diseases within 2 years after baseline in the lagged exposure analyses.

## Conclusion

In summary, our study provides substantial evidence supporting the association between a healthy sleep pattern and reduced risks of digestive diseases, regardless of genetic risk. Our findings hold considerable significance for public health efforts, underscoring the importance of enhancing comprehensive sleep traits in the prophylaxis and management of digestive diseases.

## Ethical approval

Not applicable. Data used in this study was from the UK Biobank and ethical approval was obtained from the North West Multi-Center Research Ethics Committee (Approval Numbers: 11/NW/0382, 16/NW/0274, and 21/NW/0157).

## Consent

All authors reviewed and approved the manuscript.

## Sources of funding

This work was funded by the National Natural Science Foundation of China Regional Innovation and Development Joint Foundation (U23A20408), National Natural Science Foundation of China (82171698, 82170561, 81300279, 81741067, 82273727), the Natural Science Foundation for Distinguished Young Scholars of Guangdong Province (2021B1515020003), Project to Attract Foreign Experts from Minister of Science and Technology of China (G2022030047L), Natural Science Foundation of Guangdong Province (2022A1515012081), Guangzhou Basic and Applied Basic Research Scheme -Project for Pilot Voyage (2024A04J6573), the Foreign Distinguished Teacher Program of Guangdong Science and Technology Department (KD0120220129), the Climbing Program of Introduced Talents and High-level Hospital Construction Project of Guangdong Provincial People’s Hospital (DFJH201803, KJ012019099, KJ012021143, KY012021183), and in part by VA Clinical Merit and ASGE clinical research funds (FWL).

## Author contribution

Y.Y.M., S.Y.Y., Q.M.L. and H.F.Z. contributed equally to this work. H.C., W.H.S., C.Y.D., and F.W.L. are senior and corresponding authors who also contributed equally to this study. Y.Y.M., W.H.S. and H.C. contributed to data extraction, data analyses, and manuscript drafting. S.Y.Y., Q.M.L. and H.F.Z. contributed to data interpretation and manuscript drafting. R.J.Z., R.B.L. and Q.Z.L. contributed to manuscript drafting. H.C., W.H.S., C.Y.D., and F.W.L. contributed to study design, data interpretation, and final approval of the manuscript. The corresponding author attests that all listed authors meet authorship criteria and that no others meeting the criteria have been omitted.

## Conflicts of interest disclosure

None.

## Guarantor

Prof. Hao Chen.

## Research registration unique identifying number (UIN)

This research has been conducted using the UK Biobank Resource under Application Number-83339.

## Data availability statement

Data used in this study was from the UK Biobank and are available to researchers through an access procedure described at (https://www.ukbiobank.ac.uk/enable-your-research).

## Provenance and peer review

Not commissioned, externally peer-reviewed.

## Supplementary Material
