# Supplementary material for: Sleep patterns, genetic susceptibility, and digestive diseases: a large-scale longitudinal cohort study
Source: Int J Surg. 2024 May 23;110(9):5471–82. doi: 10.1097/JS9.0000000000001695 (PMC11392193; doi:10.1097/JS9.0000000000001695)
Supplement: Supplementary file 2 [file js9-110-5471-s002.docx]

**Supplementary Figure 1. Directed Acyclic Graphs (DAG) for covariate selection.**

DAG showing the relationships between the study variables.

**
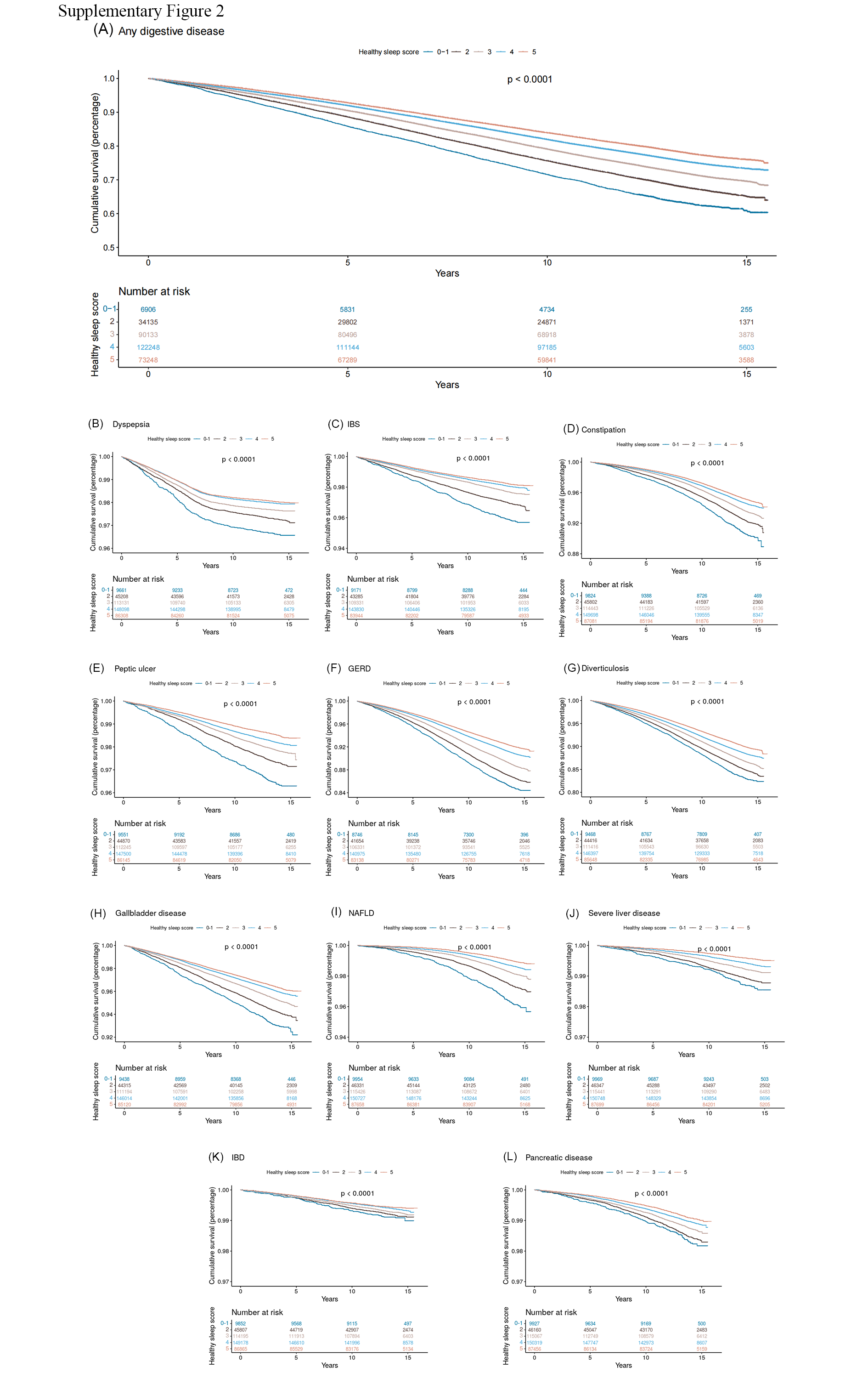
**

**Supplementary Figure 2. The Kaplan-Meier Curve for participants of different group of healthy sleep score in digestive outcomes.**

The event-free probabilities and hazard ratios as compared with participants with healthy sleep score of 0-1 are illustrated. 95% confidence intervals are shown in parentheses.

**Supplementary Materials**

***Polygenic risk score (PRS) calculations***

The PRS calculation requires a discovery sample and a target sample. The discovery sample was used to identify effect size of a set of genetic variants that were nominally associated with the disease status at a predefined P-value. The target sample included 413,164 individuals after individuals that were outliers based on heterozygosity, had a variant call rate < 99%, or being related based on a shared relatedness of up to the third degree using kinship coefficients ( > 0.044) calculated using the ukbtools packages in R. The GWAS summary statistics of IBS was derived from GERA cohort (dbGaP Study Accession: phs000674.v1.p1), NAFLD was obtained from previously published data (doi: 10.1186/s12916-019-1364-z), and other from the R9 data release of the FinnGen study were considered as discovery set for SNP selection and risk allele weighting, separately. We further excluded variants with call rate < 99%, minor allele frequency  <  0.01, deviation from Hardy–Weinberg equilibrium (P < 10^−6^) or with ambiguous strand. After LD clumping (r2 < 0.1 in 250 kb window) using the same LD reference as above, 182,790 SNPs with relative independence remained for PRS calculation. We generated PRS for each digestive outome in the target sample as the sum of the imputed SNP dosages weighted by the allele effect (logistic regression coefficients) from the discovery set across all SNPs under a P value thresholds of 1 × 10^−3^, 0.05, 0.1, 0.2, 0.3, 0.4, and 0.5, respectively. PLINK (version 1.9) was used for the PRS profiling. All scores were standardized within each target set to account for variation in SNP numbers used for PRS calculation. Subsequently, we examined the associations using logistic regression in R (version 4.2.0). Starting from a low P-value threshold moving up to P-value 0.5, an optimal P-value threshold with the highest explained variance was identified, including the most truly associated positives. We adjusted for first six PCs, sex, age, BMI and genotyping platform

**Supplementary Tables**

Supplementary Table 1. Comparison of baseline and repeated assessments data consistency

Supplementary Table 2. The Assessment of sleep behaviors

Supplementary Table 3. Outcome ascertainment

Supplemental Table 4. The baseline characteristics of excluded and included participants

Supplementary Table 5. The numbers and percentages of participants with missing covariates

Supplementary Table 6. Multivariable-adjusted HRs (95% CIs) for digestive diseases by healthy sleep score

Supplementary Table 7. HRs (95% CIs) for digestive diseases by low-risk sleep factors

Supplementary Table 8. The baseline characteristics of participants after weighting

Supplementary Table 9. HRs (95% CIs) for digestive diseases by different healthy sleep score after using inverse probability weights

Supplementary Table 10. HRs (95% CIs) for digestive diseases by different healthy sleep score in the sensitive analysis after excluding individuals who reported sleeping less than 4 hours or more than 11 hours

Supplementary Table 11. HRs (95% CIs) for digestive diseases by different healthy sleep score after excluding participants who were diagnosed with digestive diseases within 2 years after baseline

Supplementary Table 12. HRs (95% CIs) for digestive diseases in the sensitive analysis by different healthy sleep score after excluding individuals with missing covariates information

Supplementary Table 13. HRs (95% CIs) for digestive diseases by different healthy sleep score in the sensitive analysis after limiting the censoring date to December 31, 2019

Supplementary Table 14. HRs (95% CIs) for digestive diseases by different weighted sleep score

Supplementary Table 15. Subgroups analysis of healthy sleep score with digestive diseases

**Supplementary Table 1. Comparison of baseline and repeated assessments data consistency**

| **Characteristics** | **First repeat assessment visit (2012-13)** | | **Imaging visit (2014+)** | | **First repeat imaging visit (2019+)** | |
| --- | --- | --- | --- | --- | --- | --- |
|  | **N** | **Percentage** | **N** | **Percentage** | **N** | **Percentage** |
| Sleep duration | 15 422 | 76% | 43 784 | 72% | 3 701 | 70% |
| Chronotype | 14 919 | 82% | 44 311 | 80% | 3 789 | 78% |
| Insomnia | 16 160 | 80% | 45 640 | 75% | 3 886 | 74% |
| Snoring | 15 145 | 80% | 42 962 | 76% | 3 683 | 75% |
| Daytime dozing | 19 626 | 97% | 58 596 | 97% | 5 097 | 97% |

**Supplementary Table 2. The Assessment of sleep behaviors**

| **Characteristics** | **UK Biobank Code** | **UK Biobank Questionnaire** | **Healthy Answer (%)** | **Unhealthy Answer (%)** |
| --- | --- | --- | --- | --- |
| Sleep duration | 1160 | About how many hours sleep do you get in every 24 hours? (please include naps) | 7-8 hr/d. | <7 or >=9 hr/d. |
| Chronotype | 1180 | Do you consider yourself to be? | Definitely a "morning" person; More a "morning" than "evening" person. | More an "evening" than a "morning person;  Definitely an "evening" person. |
| Insomnia | 1200 | Do you have trouble falling asleep at night or do you wake up in the middle of the night? | Never/rarely: Sometimes | Usually |
| Snoring | 1210 | Does your partner or a close relative or friend complain about your snoring? | No | Yes |
| Daytime dozing | 1220 | How likely are you to doze off or fall asleep during the daytime when you don't mean to? (e.g. when working. reading or driving) | Never/rarely: Sometimes | Often: All the Time |

For further information, please refer to the UK Biobank data showcase https://biobank.ndph.ox.ac.uk/showcase/search.cgi

**Supplementary Table 3. Outcome ascertainment**

| Outcome | ICD-10 |
| --- | --- |
| Dyspepsia | K30 |
| Irritable bowel syndrome | K58 |
| Constipation | K590 |
| Peptic ulcer |  |
| Gastric ulcer | K25 |
| Duodenal ulcer | K26 |
| Other peptic ulcers | K27 |
| GERD | K21 |
| IBD |  |
| CD | K50 |
| UC | K51 |
| Gallbladder disease |  |
| Cholelithiasis | K80 |
| Cholecystitis | K81 |
| Severe liver disease | K703, K704, K721, K729, K741, 742, K746, I850, I859, K766, K767 |
| NAFLD | K758, K759, K760 |
| Pancreatic disease |  |
| Acute pancreatitis | K85 |
| Chronic pancreatitis | K861 |
| Pancreatic cyst | K862, K863 |
| Other pancreatic diseases | K868, K869 |
| Diverticulosis | K57 |

**Supplemental Table 4. The baseline characteristics of excluded and included participants**

| **Baseline characteristics** | **Excluded population** | **Included population** |
| --- | --- | --- |
|  |  |  |
| **Number of participants** | 91 782 | 410 586 |
| **Sex, female, n (%)** | 47 377 (51.6) | 225 923 (55.0) |
| **Age, mean (SD), years** | 56.8 (8.10) | 56.5 (8.09) |
| **Ethnicity, White, n (%)** | 85 115 (92.7) | 390 131 (95.0) |
| **BMI, mean (SD), kg/m^2^** | 27.6 (4.95) | 27.4 (4.77) |
| **Deprivation index, mean (SD)** | -0.79 (3.32) | -1.41 (3.03) |
| **Physical activity, mean (SD), MET minutes/week** | 2 610 (2 740) | 2 650 (2 700) |
| **Household income** |  |  |
| <18 000, n (%) | 26 826 (29.2) | 86 878 (21.2) |
| 18 000-30 999, n (%) | 24 358 (26.5) | 104 314 (25.4) |
| 31 000-51 999, n (%) | 22 042 (24.0) | 108 504 (26.4) |
| 52 000-100 000, n (%) | 14 573 (15.9) | 86 809 (21.1) |
| >100 000, n (%) | 3 983 (4.3) | 24 081 (5.9) |
| **Alcohol consumption** |  |  |
| Daily or almost daily, n (%) | 17 068 (18.6) | 85 014 (20.7) |
| Three or four times a week, n (%) | 19 311 (21.0) | 96 413 (23.5) |
| Once or twice a week, n (%) | 22 896 (24.9) | 106 766 (26.0) |
| One to three times a month, n (%) | 10 795 (11.8) | 45 258 (11.0) |
| Special occasions only or never, n (%) | 12 489 (13.6) | 45 662 (11.1) |
| Never, n (%) | 9 223 (10.0) | 31 473 (7.7) |
| **Smoking status** |  |  |
| Never smoker, n (%) | 51 195 (55.8) | 223 782 (54.5) |
| Previous smoker, n (%) | 30 070 (32.8) | 144 019 (35.1) |
| Current smoker, n (%) | 10 517 (11.5) | 42 785 (10.4) |
| **Acid inhibitor use, n (%)** | 2 795 (3.0) | 13 360 (3.3) |
| **Hospitalization, Mean (SD)** | 0.58 (1.15) | 0.53 (1.08) |
| **Comorbidities** |  |  |
| Anxiety, n (%) | 3 670 (4.0) | 15 436 (3.8) |
| Depression, n (%) | 8 397 (9.1) | 33 514 (8.2) |
| Hypertension, n (%) | 25 234 (27.5) | 108 991 (26.5) |
| Heart failure, n (%) | 588 (0.6) | 2 154 (0.5) |
| Renal failure, n (%) | 1 131 (1.2) | 4 919 (1.2) |
| Asthma, n (%) | 11 100 (12.1) | 48 906 (11.9) |
| COPD, n (%) | 2 094 (2.3) | 7 654 (1.9) |
| Diabetes, n (%) | 26 201 (28.5) | 112 425 (27.4) |

BMI: body mass index; MET: metabolic equivalent of task; COPD: chronic obstructive pulmonary disease; SD: standard deviation

**Supplementary Table 5. The numbers and percentages of participants with missing covariates**

| **Covariate** | **N** | **Percentage** |
| --- | --- | --- |
| Ethnicity | 1 131 | 0.28% |
| BMI | 2 001 | 0.49% |
| Physical activity | 70 485 | 17.17% |
| Household income | 54 914 | 13.37% |
| Education | 3 430 | 0.84% |
| Alcohol consumption | 208 | 0.05% |
| Smoking status | 1 210 | 0.29% |

BMI: body mass index

**Supplementary Table 6.** **Multivariable-adjusted HRs (95% CIs) for digestive diseases by healthy sleep score**

| **Healthy sleep score** | **N** | **Case** | **Person-year** | **Model 1** | | **Model 2** | | **Model 3** | |
| --- | --- | --- | --- | --- | --- | --- | --- | --- | --- |
|  |  |  |  | **HR (95% CI)** | **P value** | **HR (95% CI)** | **P value** | **HR (95% CI)** | **P value** |
| Any digestive disease | | | | | | | | | |
| 0-1 | 6 906 | 2 503 | 74 776.03 | 1.00 (reference) |  | 1.00 (reference) |  | 1.00 (reference) |  |
| 2 | 34 135 | 10 883 | 383 717.77 | 0.84 (0.80-0.88) | <0.01 | 0.91 (0.87-0.95) | <0.01 | 0.93 (0.89-0.98) | <0.01 |
| 3 | 90 133 | 25 162 | 1 044 024.02 | 0.71 (0.68-0.74) | <0.01 | 0.82 (0.78-0.85) | <0.01 | 0.85 (0.81-0.88) | <0.01 |
| 4 | 122 248 | 29 864 | 1 451 418.29 | 0.61 (0.59-0.63) | <0.01 | 0.74 (0.71-0.77) | <0.01 | 0.77 (0.74-0.81) | <0.01 |
| 5 | 73 248 | 16 095 | 884 445.96 | 0.54 (0.52-0.56) | <0.01 | 0.68 (0.65-0.71) | <0.01 | 0.72 (0.69-0.75) | <0.01 |
| Dyspepsia | | | | | | | | | |
| 0-1 | 9 661 | 316 | 124 023.82 | 1.00 (reference) |  | 1.00 (reference) |  | 1.00 (reference) |  |
| 2 | 45 208 | 1 198 | 587 312.33 | 0.79 (0.70-0.90) | <0.01 | 0.83 (0.73-0.94) | <0.01 | 0.85 (0.75-0.96) | 0.01 |
| 3 | 113 131 | 2 584 | 1 481 181.97 | 0.68 (0.61-0.77) | <0.01 | 0.74 (0.66-0.83) | <0.01 | 0.77 (0.68-0.86) | <0.01 |
| 4 | 148 098 | 2 953 | 1 951 808.78 | 0.59 (0.53-0.67) | <0.01 | 0.66 (0.58-0.74) | <0.01 | 0.69 (0.62-0.78) | <0.01 |
| 5 | 86 308 | 1 661 | 1 141 993.44 | 0.55 (0.49-0.63) | <0.01 | 0.62 (0.55-0.70) | <0.01 | 0.66 (0.58-0.75) | <0.01 |
| IBS | | | | | | | | | |
| 0-1 | 9 171 | 361 | 117 785.42 | 1.00 (reference) |  | 1.00 (reference) |  | 1.00 (reference) |  |
| 2 | 43 285 | 1 269 | 562 089.46 | 0.73 (0.65-0.82) | <0.01 | 0.77 (0.69-0.87) | <0.01 | 0.80 (0.71-0.90) | <0.01 |
| 3 | 109 331 | 2 439 | 1 434 012.19 | 0.55 (0.49-0.62) | <0.01 | 0.61 (0.55-0.69) | <0.01 | 0.65 (0.59-0.73) | <0.01 |
| 4 | 143 830 | 2 710 | 1 898 342.05 | 0.45 (0.41-0.51) | <0.01 | 0.52 (0.47-0.59) | <0.01 | 0.57 (0.51-0.64) | <0.01 |
| 5 | 83 944 | 1 454 | 1 113 408.00 | 0.39 (0.35-0.44) | <0.01 | 0.46 (0.41-0.51) | <0.01 | 0.50 (0.45-0.57) | <0.01 |
| Constipation | | | | | | | | | |
| 0-1 | 9 824 | 856 | 124 947.33 | 1.00 (reference) |  | 1.00 (reference) |  | 1.00 (reference) |  |
| 2 | 45 802 | 3 281 | 591 007.12 | 0.80 (0.74-0.86) | <0.01 | 0.89 (0.82-0.96) | <0.01 | 0.92 (0.85-0.99) | 0.03 |
| 3 | 114 443 | 6 892 | 1 492 151.05 | 0.66 (0.61-0.71) | <0.01 | 0.79 (0.73-0.85) | <0.01 | 0.84 (0.78-0.90) | <0.01 |
| 4 | 149 698 | 7 634 | 1 965 878.95 | 0.56 (0.52-0.60) | <0.01 | 0.71 (0.66-0.76) | <0.01 | 0.76 (0.71-0.82) | <0.01 |
| 5 | 87 081 | 4 012 | 1 149 703.15 | 0.51 (0.47-0.55) | <0.01 | 0.66 (0.62-0.72) | <0.01 | 0.72 (0.67-0.78) | <0.01 |
| Peptic ulcer | | | | | | | | | |
| 0-1 | 9 551 | 325 | 123 205.25 | 1.00 (reference) |  | 1.00 (reference) |  | 1.00 (reference) |  |
| 2 | 44 870 | 1 149 | 586 106.63 | 0.74 (0.65-0.84) | <0.01 | 0.85 (0.75-0.96) | <0.01 | 0.87 (0.77-0.99) | 0.03 |
| 3 | 112 245 | 2 321 | 1 477 977.41 | 0.59 (0.52-0.66) | <0.01 | 0.74 (0.66-0.83) | <0.01 | 0.78 (0.69-0.88) | <0.01 |
| 4 | 147 500 | 2 577 | 1 953 065.87 | 0.50 (0.45-0.56) | <0.01 | 0.67 (0.60-0.76) | <0.01 | 0.72 (0.64-0.81) | <0.01 |
| 5 | 86 145 | 1 248 | 1 146 491.75 | 0.43 (0.38-0.48) | <0.01 | 0.61 (0.54-0.69) | <0.01 | 0.65 (0.58-0.74) | <0.01 |
| Gastric ulcer | | | | | | | | | |
| 0-1 | 9 744 | 229 | 126 305.06 | 1.00 (reference) |  | 1.00 (reference) |  | 1.00 (reference) |  |
| 2 | 45 609 | 836 | 597 392.38 | 0.77 (0.66-0.89) | <0.01 | 0.88 (0.76-1.02) | 0.09 | 0.91 (0.78-1.05) | 0.20 |
| 3 | 113 855 | 1 640 | 1 502 817.25 | 0.60 (0.52-0.68) | <0.01 | 0.75 (0.65-0.86) | <0.01 | 0.79 (0.69-0.91) | <0.01 |
| 4 | 149 185 | 1 796 | 1 979 848.34 | 0.50 (0.44-0.58) | <0.01 | 0.68 (0.59-0.78) | <0.01 | 0.73 (0.63-0.83) | <0.01 |
| 5 | 86 948 | 885 | 1 159 301.20 | 0.43 (0.37-0.50) | <0.01 | 0.61 (0.53-0.71) | <0.01 | 0.66 (0.57-0.77) | <0.01 |
| Duodenal ulcer | | | | | | | | | |
| 0-1 | 9 769 | 106 | 127 426.02 | 1.00 (reference) |  | 1.00 (reference) |  | 1.00 (reference) |  |
| 2 | 45 629 | 419 | 600 244.85 | 0.84 (0.68-1.04) | 0.11 | 0.96 (0.78-1.19) | 0.71 | 0.99 (0.80-1.23) | 0.93 |
| 3 | 113 878 | 895 | 1 507 696.94 | 0.71 (0.58-0.86) | <0.01 | 0.89 (0.72-1.08) | 0.24 | 0.93 (0.76-1.14) | 0.49 |
| 4 | 149 099 | 989 | 1 983 603.72 | 0.61 (0.50-0.74) | <0.01 | 0.82 (0.67-1.00) | 0.05 | 0.87 (0.71-1.07) | 0.18 |
| 5 | 86 930 | 433 | 1 161 660.54 | 0.49 (0.39-0.60) | <0.01 | 0.69 (0.56-0.86) | <0.01 | 0.74 (0.60-0.92) | <0.01 |
| Other peptic ulcers | | | | | | | | | |
| 0-1 | 9 951 | 36 | 130 141.68 | 1.00 (reference) |  | 1.00(reference) |  | 1.00(reference) |  |
| 2 | 46 273 | 87 | 610 597.96 | 0.51 (0.35-0.76) | <0.01 | 0.62 (0.42-0.92) | 0.02 | 0.66 (0.45-0.98) | 0.04 |
| 3 | 115 230 | 151 | 1 529 392.96 | 0.35 (0.24-0.51) | <0.01 | 0.49 (0.34-0.71) | <0.01 | 0.54 (0.37-0.78) | <0.01 |
| 4 | 150 555 | 129 | 2 007 672.22 | 0.23 (0.16-0.34) | <0.01 | 0.36 (0.25-0.53) | <0.01 | 0.41 (0.28-0.60) | <0.01 |
| 5 | 87 566 | 83 | 1 171 992.19 | 0.27 (0.18-0.40) | <0.01 | 0.45 (0.3-0.68) | <0.01 | 0.51 (0.34-0.77) | <0.01 |
| GERD | | | | | | | | | |
| 0-1 | 8 746 | 1 234 | 107 069.80 | 1.00 (reference) |  | 1.00 (reference) |  | 1.00 (reference) |  |
| 2 | 41 654 | 5 230 | 518 823.17 | 0.87 (0.82-0.92) | <0.01 | 0.95 (0.89-1.01) | 0.09 | 0.98 (0.92-1.04) | 0.52 |
| 3 | 106 331 | 11 175 | 1 346 848.20 | 0.71 (0.67-0.76) | <0.01 | 0.83 (0.78-0.88) | <0.01 | 0.88 (0.82-0.93) | <0.01 |
| 4 | 140 975 | 12 136 | 1 809 856.60 | 0.58 (0.55-0.61) | <0.01 | 0.71 (0.67-0.75) | <0.01 | 0.76 (0.72-0.81) | <0.01 |
| 5 | 83 138 | 6 247 | 1 076 582.34 | 0.50 (0.47-0.53) | <0.01 | 0.63 (0.60-0.68) | <0.01 | 0.68 (0.64-0.73) | <0.01 |
| IBD | | | | | | | | | |
| 0-1 | 9 852 | 85 | 128 565.25 | 1.00 (reference) |  | 1.00 (reference) |  | 1.00 (reference) |  |
| 2 | 45 807 | 358 | 602 832.56 | 0.90 (0.71-1.14) | 0.37 | 1.00 (0.79-1.27) | 0.99 | 1.02 (0.80-1.29) | 0.90 |
| 3 | 114 195 | 801 | 1 511 787.02 | 0.80 (0.64-1.00) | 0.05 | 0.96 (0.77-1.21) | 0.76 | 0.98 (0.79-1.23) | 0.89 |
| 4 | 149 178 | 894 | 1 984 615.31 | 0.68 (0.55-0.86) | <0.01 | 0.88 (0.70-1.10) | 0.26 | 0.90 (0.72-1.13) | 0.36 |
| 5 | 86 865 | 479 | 1 160 111.36 | 0.64 (0.51-0.81) | <0.01 | 0.86 (0.68-1.09) | 0.21 | 0.88 (0.70-1.12) | 0.30 |
| Crohn’s disease | | | | | | | | | |
| 0-1 | 9 936 | 41 | 129 935.01 | 1.00 (reference) |  | 1.00 (reference) |  | 1.00 (reference) |  |
| 2 | 46 209 | 146 | 609 315.08 | 0.76 (0.54-1.07) | 0.12 | 0.87 (0.61-1.23) | 0.42 | 0.88 (0.62-1.25) | 0.48 |
| 3 | 115 098 | 327 | 1 526 646.10 | 0.68 (0.49-0.94) | 0.02 | 0.85 (0.61-1.18) | 0.33 | 0.87 (0.63-1.21) | 0.41 |
| 4 | 150 258 | 364 | 2 002 287.01 | 0.58 (0.42-0.80) | <0.01 | 0.78 (0.56-1.08) | 0.13 | 0.80 (0.58-1.11) | 0.19 |
| 5 | 87 417 | 206 | 1 169 272.66 | 0.56 (0.40-0.78) | <0.01 | 0.79 (0.56-1.11) | 0.18 | 0.82 (0.58-1.15) | 0.25 |
| Ulcerative colitis | | | | | | | | | |
| 0-1 | 9 890 | 59 | 129 215.97 | 1.00 (reference) |  | 1.00 (reference) |  | 1.00 (reference) |  |
| 2 | 45 966 | 259 | 605 514.75 | 0.94 (0.71-1.24) | 0.66 | 1.04 (0.78-1.38) | 0.79 | 1.04 (0.79-1.39) | 0.76 |
| 3 | 114 582 | 604 | 1 517 968.40 | 0.87 (0.67-1.14) | 0.30 | 1.04 (0.79-1.36) | 0.79 | 1.05 (0.80-1.37) | 0.73 |
| 4 | 149 658 | 684 | 1 992 140.21 | 0.76 (0.58-0.99) | 0.04 | 0.96 (0.73-1.25) | 0.75 | 0.97 (0.74-1.27) | 0.82 |
| 5 | 87 132 | 361 | 1 164 352.69 | 0.70 (0.53-0.92) | 0.01 | 0.93 (0.70-1.22) | 0.59 | 0.94 (0.71-1.24) | 0.65 |
| Gallbladder disease | | | | | | | | | |
| 0-1 | 9 438 | 624 | 119 713.19 | 1.00 (reference) |  | 1.00 (reference) |  | 1.00 (reference) |  |
| 2 | 44 315 | 2 464 | 570 315.62 | 0.82 (0.75-0.89) | <0.01 | 0.97 (0.89-1.06) | 0.56 | 0.99 (0.91-1.08) | 0.86 |
| 3 | 111 194 | 5 081 | 1 446 173.31 | 0.67 (0.61-0.72) | <0.01 | 0.89 (0.82-0.97) | <0.01 | 0.92 (0.84-1.00) | 0.05 |
| 4 | 146 014 | 5 658 | 1 913 380.10 | 0.56 (0.52-0.61) | <0.01 | 0.82 (0.76-0.89) | <0.01 | 0.85 (0.78-0.93) | <0.01 |
| 5 | 85 120 | 3 020 | 1 121 380.27 | 0.50 (0.46-0.54) | <0.01 | 0.79 (0.72-0.86) | <0.01 | 0.82 (0.75-0.90) | <0.01 |
| Cholelithiasis | | | | | | | | | |
| 0-1 | 9 471 | 577 | 120 420.48 | 1.00 (reference) |  | 1.00 (reference) |  | 1.00 (reference) |  |
| 2 | 44 460 | 2 273 | 573 184.86 | 0.82 (0.75-0.90) | <0.01 | 0.97 (0.89-1.07) | 0.56 | 0.99 (0.90-1.09) | 0.84 |
| 3 | 111 484 | 4 629 | 1 452 431.41 | 0.66 (0.60-0.72) | <0.01 | 0.88 (0.81-0.96) | <0.01 | 0.91 (0.83-0.99) | 0.03 |
| 4 | 146 354 | 5 221 | 1 920 191.33 | 0.56 (0.51-0.61) | <0.01 | 0.82 (0.76-0.90) | <0.01 | 0.86 (0.78-0.93) | <0.01 |
| 5 | 85 303 | 2 762 | 1 125 152.43 | 0.49 (0.45-0.54) | <0.01 | 0.78 (0.71-0.86) | <0.01 | 0.82 (0.74-0.89) | <0.01 |
| Cholecystitis | | | | | | | | | |
| 0-1 | 9 877 | 117 | 128 713.52 | 1.00 (reference) |  | 1.00 (reference) |  | 1.00 (reference) |  |
| 2 | 46 057 | 469 | 605 480.72 | 0.85 (0.69-1.04) | 0.11 | 1.02 (0.83-1.25) | 0.84 | 1.04 (0.85-1.28) | 0.69 |
| 3 | 114 858 | 989 | 1 519 387.43 | 0.71 (0.59-0.86) | <0.01 | 0.97 (0.80-1.18) | 0.76 | 1.01 (0.83-1.22) | 0.96 |
| 4 | 150 065 | 982 | 1 996 032.01 | 0.54 (0.45-0.66) | <0.01 | 0.81 (0.67-0.99) | 0.04 | 0.85 (0.70-1.03) | 0.11 |
| 5 | 87 316 | 571 | 1 165 746.93 | 0.55 (0.45-0.67) | <0.01 | 0.89 (0.73-1.09) | 0.25 | 0.94 (0.76-1.15) | 0.52 |
| Severe liver disease | | | | | | | | | |
| 0-1 | 9 969 | 126 | 130 264.84 | 1.00 (reference) |  | 1.00 (reference) |  | 1.00 (reference) |  |
| 2 | 46 347 | 492 | 610 639.85 | 0.84 (0.69-1.02) | 0.07 | 1.07 (0.88-1.30) | 0.51 | 1.11 (0.91-1.35) | 0.30 |
| 3 | 115 441 | 891 | 1 530 678.69 | 0.60 (0.49-0.72) | <0.01 | 0.91 (0.75-1.10) | 0.32 | 0.97 (0.80-1.17) | 0.74 |
| 4 | 150 748 | 881 | 2 008 812.42 | 0.46 (0.38-0.55) | <0.01 | 0.79 (0.66-0.96) | 0.02 | 0.86 (0.71-1.04) | 0.13 |
| 5 | 87 699 | 368 | 1 173 246.42 | 0.36 (0.29-0.43) | <0.01 | 0.69 (0.56-0.85) | <0.01 | 0.76 (0.62-0.93) | <0.01 |
| NAFLD | | | | | | | | | |
| 0-1 | 9 954 | 342 | 128 919.05 | 1.00 (reference) |  | 1.00 (reference) |  | 1.00 (reference) |  |
| 2 | 46 331 | 1 123 | 607 073.99 | 0.69 (0.61-0.78) | <0.01 | 0.92 (0.82-1.04) | 0.19 | 0.96 (0.85-1.08) | 0.48 |
| 3 | 115 426 | 1 932 | 1 524 890.72 | 0.47 (0.42-0.53) | <0.01 | 0.77 (0.68-0.86) | <0.01 | 0.82 (0.73-0.92) | <0.01 |
| 4 | 150 727 | 1 895 | 2 003 445.33 | 0.35 (0.32-0.40) | <0.01 | 0.67 (0.59-0.75) | <0.01 | 0.72 (0.64-0.81) | <0.01 |
| 5 | 87 658 | 825 | 1 170 668.22 | 0.27 (0.23-0.30) | <0.01 | 0.57 (0.50-0.65) | <0.01 | 0.63 (0.55-0.71) | <0.01 |
| Pancreatic disease | | | | | | | | | |
| 0-1 | 9 927 | 153 | 129 433.96 | 1.00 (reference) |  | 1.00 (reference) |  | 1.00 (reference) |  |
| 2 | 46 160 | 653 | 606 706.46 | 0.90 (0.76-1.08) | 0.25 | 1.06 (0.88-1.26) | 0.19 | 1.08 (0.91-1.29) | 0.38 |
| 3 | 115 067 | 1 349 | 1 522 259.55 | 0.74 (0.62-0.87) | <0.01 | 0.96 (0.81-1.14) | <0.01 | 1.00 (0.84-1.18) | 1.00 |
| 4 | 150 319 | 1 471 | 1 998 949.95 | 0.62 (0.53-0.73) | <0.01 | 0.88 (0.74-1.04) | <0.01 | 0.92 (0.78-1.09) | 0.34 |
| 5 | 87 456 | 748 | 1 167 828.46 | 0.55 (0.46-0.65) | <0.01 | 0.83 (0.69-0.99) | <0.01 | 0.87 (0.73-1.04) | 0.13 |
| Acute pancreatitis | | | | | | | | | |
| 0-1 | 9 932 | 85 | 129 696.68 | 1.00 (reference) |  | 1.00 (reference) |  | 1.00 (reference) |  |
| 2 | 46 181 | 368 | 607 851.78 | 0.92 (0.73-1.16) | 0.48 | 1.11 (0.88-1.41) | 0.37 | 1.14 (0.90-1.44) | 0.29 |
| 3 | 115 104 | 758 | 1 524 532.84 | 0.75 (0.60-0.94) | 0.01 | 1.04 (0.83-1.30) | 0.73 | 1.07 (0.85-1.34) | 0.55 |
| 4 | 150 365 | 823 | 2 001 705.08 | 0.63 (0.50-0.79) | <0.01 | 0.96 (0.77-1.20) | 0.72 | 1.00 (0.79-1.25) | 0.98 |
| 5 | 87 480 | 414 | 1 169 214.83 | 0.55 (0.44-0.69) | <0.01 | 0.91 (0.72-1.16) | 0.44 | 0.95 (0.75-1.21) | 0.67 |
| Chronic pancreatitis | | | | | | | | | |
| 0-1 | 9 978 | 24 | 130 661.63 | 1.00 (reference) |  | 1.00 (reference) |  | 1.00 (reference) |  |
| 2 | 46 388 | 118 | 612 024.48 | 1.05 (0.68-1.63) | 0.82 | 1.30 (0.84-2.02) | 0.24 | 1.37 (0.88-2.12) | 0.16 |
| 3 | 115 524 | 195 | 1 533 183.57 | 0.69 (0.45-1.05) | 0.08 | 0.99 (0.64-1.52) | 0.96 | 1.07 (0.70-1.64) | 0.76 |
| 4 | 150 820 | 213 | 2 011 067.63 | 0.58 (0.38-0.89) | 0.01 | 0.94 (0.61-1.44) | 0.77 | 1.03 (0.67-1.59) | 0.88 |
| 5 | 87 718 | 86 | 1 174 184.95 | 0.44 (0.28-0.69) | <0.01 | 0.76 (0.48-1.20) | 0.24 | 0.84 (0.53-1.34) | 0.47 |
| Pancreatic cyst | | | | | | | | | |
| 0-1 | 9 982 | 24 | 130 689.83 | 1.00 (reference) |  | 1.00 (reference) |  | 1.00 (reference) |  |
| 2 | 46 410 | 125 | 612 337.01 | 1.1 (0.71-1.70) | 0.68 | 1.19 (0.77-1.85) | 0.43 | 1.22 (0.79-1.89) | 0.37 |
| 3 | 115 558 | 246 | 1 533 572.57 | 0.86 (0.56-1.30) | 0.47 | 0.99 (0.65-1.51) | 0.97 | 1.02 (0.67-1.56) | 0.92 |
| 4 | 150 847 | 291 | 2 010 933.96 | 0.78 (0.52-1.18) | 0.25 | 0.95 (0.62-1.44) | 0.79 | 0.98 (0.64-1.50) | 0.94 |
| 5 | 87 723 | 149 | 1 173 916.70 | 0.69 (0.45-1.06) | 0.09 | 0.84 (0.55-1.31) | 0.45 | 0.88 (0.57-1.37) | 0.58 |
| Other pancreatic diseases | | | | | | | | | |
| 0-1 | 9 978 | 54 | 130 556.78 | 1.00 (reference) |  | 1.00 (reference) |  | 1.00 (reference) |  |
| 2 | 46 408 | 231 | 612 002.47 | 0.90 (0.67-1.21) | 0.49 | 1.03 (0.77-1.39) | 0.49 | 1.06 (0.78-1.42) | 0.72 |
| 3 | 115 555 | 502 | 1 532 960.53 | 0.77 (0.58-1.03) | 0.07 | 0.97 (0.73-1.29) | 0.07 | 1.01 (0.76-1.34) | 0.95 |
| 4 | 150 849 | 507 | 2 010 639.55 | 0.61 (0.46-0.80) | <0.01 | 0.82 (0.62-1.09) | <0.01 | 0.86 (0.64-1.14) | 0.28 |
| 5 | 87 729 | 253 | 1 173 856.63 | 0.53 (0.39-0.71) | <0.01 | 0.75 (0.56-1.01) | <0.01 | 0.79 (0.58-1.06) | 0.12 |
| Diverticulosis | | | | | | | | | |
| 0-1 | 9 468 | 1 508 | 114 995.55 | 1.00 (reference) |  | 1.00 (reference) |  | 1.00 (reference) |  |
| 2 | 44 416 | 6 361 | 549 067.90 | 0.87 (0.82-0.92) | <0.01 | 0.94 (0.89-1.00) | 0.03 | 0.96 (0.91-1.02) | 0.18 |
| 3 | 111 416 | 14 067 | 1 397 468.97 | 0.75 (0.71-0.79) | <0.01 | 0.86 (0.81-0.91) | <0.01 | 0.89 (0.84-0.94) | <0.01 |
| 4 | 146 397 | 16 128 | 1 858 742.27 | 0.65 (0.62-0.69) | <0.01 | 0.79 (0.74-0.83) | <0.01 | 0.82 (0.78-0.87) | <0.01 |
| 5 | 85 648 | 8 244 | 1 099 421.36 | 0.57 (0.54-0.60) | <0.01 | 0.72 (0.68-0.76) | <0.01 | 0.75 (0.71-0.80) | <0.01 |

IBS: irritable bowel syndrome; GERD: gastroesophageal reflux disease; IBD: inflammatory bowel disease; NAFLD: nonalcoholic fatty liver disease; HR: hazard ratio; CI: confidence interval.

Model 1: adjusted for age and sex.

Model 2: adjusted for covariates in model 1 and ethnicity, body mass index (BMI), Townsend Deprivation Index, household income, education, acid inhibitor use, the number of hospital admissions three years before the baseline, smoking status, alcohol consumption and physical activity.

Model 3: adjusted for covariates in model 2 and comorbidities, including hypertension, heart failure, MI, stroke, asthma, renal failure, COPD, thyroid disease, anxiety, depression, dementia, and diabetes.

**Supplementary Table 7. HRs (95% CIs) for digestive diseases by low-risk sleep factors**

| **Low-risk sleep factors** | **N** | **Case** | **HR (95% CI) *** | **P value** |
| --- | --- | --- | --- | --- |
| Any digestive diseases |  |  |  |  |
| Never/rarely insomnia | 242 299 | 58 455 | 0.84(0.83-0.86) | <0.01 |
| Early chronotype | 206 208 | 52 666 | 0.94(0.93-0.96) | <0.01 |
| No frequent daytime sleepiness | 318 554 | 81 714 | 0.85(0.82-0.88) | <0.01 |
| Sleep 7-8 h/day | 226 584 | 55 541 | 0.90(0.89-0.92) | <0.01 |
| No self-reported snoring | 206 712 | 51 112 | 0.95(0.94-0.96) | <0.01 |
| All five factors | 73 248 | 84 507 | 0.87(0.86-0.89) | <0.01 |
| Dyspepsia |  |  |  |  |
| Never/rarely insomnia | 291 265 | 5 687 | 0.82(0.78-0.86) | <0.01 |
| Early chronotype | 252 514 | 5 404 | 0.94(0.90-0.99) | <0.01 |
| No frequent daytime sleepiness | 391 469 | 8 396 | 0.86(0.77-0.96) | <0.01 |
| Sleep 7-8 h/day | 274 624 | 5 520 | 0.88(0.84-0.92) | <0.01 |
| No self-reported snoring | 252 857 | 5 551 | 0.97(0.93-1.02) | 0.21 |
| All five factors | 86 308 | 8 712 | 0.88(0.83-0.93) | <0.01 |
| IBS |  |  |  |  |
| Never/rarely insomnia | 283 239 | 5 089 | 0.71(0.68-0.74) | <0.01 |
| Early chronotype | 245 193 | 4 921 | 0.90(0.86-0.94) | <0.01 |
| No frequent daytime sleepiness | 379 084 | 7 856 | 0.65(0.59-0.73) | <0.01 |
| Sleep 7-8 h/day | 266 329 | 5 100 | 0.82(0.78-0.86) | <0.01 |
| No self-reported snoring | 244 297 | 5 323 | 0.97(0.93-1.02) | 0.26 |
| All five factors | 83 944 | 8 233 | 0.79(0.74-0.83) | <0.01 |
| Constipation |  |  |  |  |
| Never/rarely insomnia | 294 153 | 14 715 | 0.84(0.81-0.86) | <0.01 |
| Early chronotype | 255 318 | 14 036 | 0.93(0.91-0.96) | <0.01 |
| No frequent daytime sleepiness | 395 794 | 21 634 | 0.76(0.71-0.81) | <0.01 |
| Sleep 7-8 h/day | 277 506 | 14 072 | 0.88(0.85-0.90) | <0.01 |
| No self-reported snoring | 255 502 | 14 148 | 1.04(1.01-1.07) | <0.01 |
| All five factors | 87 081 | 22 675 | 0.88(0.85-0.91) | <0.01 |
| Peptic ulcer |  |  |  |  |
| Never/rarely insomnia | 289 820 | 4 945 | 0.81(0.77-0.85) | <0.01 |
| Early chronotype | 251 303 | 4 685 | 0.94(0.89-0.98) | <0.01 |
| No frequent daytime sleepiness | 389 485 | 7 284 | 0.84(0.75-0.94) | <0.01 |
| Sleep 7-8 h/day | 273 543 | 4 648 | 0.85(0.82-0.90) | <0.01 |
| No self-reported snoring | 251 937 | 4 549 | 1.00(0.96-1.05) | 0.92 |
| All five factors | 86 145 | 7 620 | 0.85(0.80-0.90) | <0.01 |
| GERD |  |  |  |  |
| Never/rarely insomnia | 277 646 | 23 517 | 0.79(0.77-0.81) | <0.01 |
| Early chronotype | 239 546 | 22 300 | 0.95(0.93-0.97) | <0.01 |
| No frequent daytime sleepiness | 370 780 | 34 686 | 0.85(0.80-0.90) | <0.01 |
| Sleep 7-8 h/day | 261 149 | 22 804 | 0.88(0.86-0.90) | <0.01 |
| No self-reported snoring | 240 932 | 21 591 | 0.91(0.89-0.93) | <0.01 |
| All five factors | 83 138 | 36 022 | 0.82(0.80-0.84) | <0.01 |
| IBD |  |  |  |  |
| Never/rarely insomnia | 293 281 | 1 796 | 0.93(0.85-1.01) | 0.09 |
| Early chronotype | 254 702 | 1 581 | 0.93(0.86-1.00) | 0.06 |
| No frequent daytime sleepiness | 394 811 | 2 518 | 0.88(0.72-1.08) | 0.23 |
| Sleep 7-8 h/day | 276 628 | 1 616 | 0.84(0.78-0.91) | <0.01 |
| No self-reported snoring | 254 981 | 1 652 | 1.12(1.03-1.21) | <0.01 |
| All five factors | 86 865 | 2 617 | 0.93(0.84-1.03) | 0.16 |
| Gallbladder disease |  |  |  |  |
| Never/rarely insomnia | 287 158 | 11 172 | 0.90(0.87-0.93) | <0.01 |
| Early chronotype | 248 586 | 10 205 | 0.91(0.88-0.94) | <0.01 |
| No frequent daytime sleepiness | 385 331 | 16 266 | 1.04(0.96-1.13) | 0.32 |
| Sleep 7-8 h/day | 270 619 | 10 770 | 0.96(0.93-0.99) | <0.01 |
| No self-reported snoring | 248 947 | 10 069 | 0.97(0.94-1.00) | 0.03 |
| All five factors | 85 120 | 16 847 | 0.91(0.88-0.95) | <0.01 |
| Severe liver disease |  |  |  |  |
| Never/rarely insomnia | 296 166 | 1 744 | 0.81(0.75-0.88) | <0.01 |
| Early chronotype | 257 367 | 1 617 | 0.90(0.84-0.98) | 0.01 |
| No frequent daytime sleepiness | 398 967 | 2 636 | 1.02(0.85-1.23) | 0.82 |
| Sleep 7-8 h/day | 279 535 | 1 549 | 0.77(0.71-0.83) | <0.01 |
| No self-reported snoring | 257 736 | 1 590 | 1.10(1.01-1.19) | 0.02 |
| All five factors | 87 699 | 2 758 | 0.80(0.72-0.90) | <0.01 |
| NAFLD |  |  |  |  |
| Never/rarely insomnia | 296 096 | 3 834 | 0.82(0.78-0.87) | <0.01 |
| Early chronotype | 257 294 | 3 632 | 0.92(0.88-0.97) | <0.01 |
| No frequent daytime sleepiness | 398 855 | 5 787 | 0.82(0.73-0.91) | <0.01 |
| Sleep 7-8 h/day | 279 446 | 3 578 | 0.86(0.81-0.90) | <0.01 |
| No self-reported snoring | 257 702 | 3 222 | 0.86(0.82-0.91) | <0.01 |
| All five factors | 87 658 | 6 117 | 0.78(0.72-0.84) | <0.01 |
| Pancreatic disease |  |  |  |  |
| Never/rarely insomnia | 295 329 | 2 848 | 0.85(0.80-0.91) | <0.01 |
| Early chronotype | 256 573 | 2 699 | 0.95(0.89-1.01) | 0.07 |
| No frequent daytime sleepiness | 397 731 | 4 215 | 1.05(0.89-1.23) | 0.57 |
| Sleep 7-8 h/day | 278 732 | 2 735 | 0.92(0.86-0.98) | <0.01 |
| No self-reported snoring | 256 942 | 2 613 | 0.99(0.93-1.05) | 0.77 |
| All five factors | 87 456 | 4 374 | 0.89(0.82-0.97) | <0.01 |
| Diverticulosis |  |  |  |  |
| Never/rarely insomnia | 288 150 | 31 177 | 0.87(0.85-0.89) | <0.01 |
| Early chronotype | 249 324 | 28 867 | 0.95(0.94-0.97) | <0.01 |
| No frequent daytime sleepiness | 386 631 | 44 756 | 0.93(0.88-0.98) | <0.01 |
| Sleep 7-8 h/day | 271 478 | 30 112 | 0.93(0.91-0.95) | <0.01 |
| No self-reported snoring | 250 128 | 27 132 | 0.91(0.90-0.93) | <0.01 |
| All five factors | 85 648 | 46 308 | 0.87(0.85-0.89) | <0.01 |

IBS: irritable bowel syndrome; GERD: gastroesophageal reflux disease; IBD: inflammatory bowel disease; NAFLD: nonalcoholic fatty liver disease; HR: hazard ratio; CI: confidence interval.

*Adjusted for age and sex, ethnicity, body mass index, Townsend Deprivation Index, household income, education, acid inhibitor use, the number of hospital admissions three years before the baseline, smoking status, alcohol consumption, physical activity, hypertension, heart failure, MI, stroke, asthma, renal failure, COPD, thyroid disease, anxiety, depression, dementia, and diabetes.

**Supplementary Table 8.** **The baseline characteristics of participants after weighting**

| **Baseline characteristics** | **Healthy sleep score** | | | | | **SMD** |
| --- | --- | --- | --- | --- | --- | --- |
|  | **0-1** | **2** | **3** | **4** | **5** |  |
| **Number of participants, n** | 7 216.8 | 45095.5 | 117 341.8 | 151 890 | 90 698.5 |  |
| **Sex, female, n (%)** | 3 945.7 (54.7) | 24 837.8 (55.1) | 64 385.9 (54.9) | 83 394.6 (54.9) | 49 621.8 (54.7) | 0.004 |
| **Age, mean (SD), years** | 56.8 (7.6) | 56.7 (7.8) | 56.6 (8.0) | 56.5 (8.2) | 56.6 (8.2) | 0.017 |
| **Ethnicity, White, n (%)** | 6 757.9 (93.6) | 42 982.0 (95.3) | 111 266.7 (94.8) | 144 392.2 (95.1) | 86 102.6 (94.9) | 0.032 |
| **BMI, mean (SD), kg/m2** | 27.8 (4.9) | 27.5 (4.8) | 27.4 (4.7) | 27.4 (4.8) | 27.5 (5.1) | 0.033 |
| **Deprivation index, mean (SD)** | -1.3 (3.1) | -1.4 (3.0) | -1.4 (3.0) | -1.4 (3.0) | -1.4 (3.1) | 0.020 |
| **Physical activity, mean (SD), MET minutes/week** | 2 746.0 (3 068.1) | 2 748.9 (2 940.2) | 2 608.3 (2 710.2) | 2 623.7 (2 646.6) | 2 716.3 (2 662.3) | 0.029 |
| **Household income** |  |  |  |  |  | 0.074 |
| <18 000, n (%) | 1 753.1 (24.3) | 9 450.3 (21.0) | 24 674.8 (21.0) | 32 516.0 (21.4) | 19 770.7 (21.8) |  |
| 18 000-30 999, n (%) | 1 839.2 (25.5) | 11 557.0 (25.6) | 30 313.7 (25.8) | 38 770.3 (25.5) | 22 427.8 (24.7) |  |
| 31 000-51 999, n (%) | 2 044.7 (28.3) | 11 857.3 (26.3) | 30 608.5 (26.1) | 39 716.9 (26.1) | 24 338.6 (26.8) |  |
| 52 000-100 000, n (%) | 1 332.5 (18.5) | 9 709.3 (21.5) | 24 959.3 (21.3) | 31 990.7 (21.1) | 18 611.5 (20.5) |  |
| >100 000, n (%) | 247.4 (3.4) | 2 521.5 (5.6) | 6 785.6 (5.8) | 8 896.0 (5.9) | 5 549.9 (6.1) |  |
| **Alcohol consumption** |  |  |  |  |  | 0.058 |
| Daily or almost daily, n (%) | 1 708.4 (23.7) | 9 451.6 (21.0) | 24 282.6 (20.7) | 31 032.5 (20.4) | 18 869.2 (20.8) |  |
| Three or four times a week, n (%) | 1 741.9 (24.1) | 10 457.3 (23.2) | 27 503.0 (23.4) | 35 615.0 (23.4) | 20 989.6 (23.1) |  |
| Once or twice a week, n (%) | 1 705.2 (23.6) | 11 616.7 (25.8) | 30 556.1 (26.0) | 39 694.9 (26.1) | 23 458.3 (25.9) |  |
| One to three times a month, n (%) | 758.4 (10.5) | 5 288.1 (11.7) | 12 524.2 (10.7) | 16 256.0 (10.7) | 10 585.9 (11.7) |  |
| Special occasions only or never, n (%) | 748.2 (10.4) | 5 012.3 (11.1) | 13 741.6 (11.7) | 17 023.2 (11.2) | 9 588.9 (10.6) |  |
| Never, n (%) | 554.7 (7.7) | 3 269.5 (7.3) | 8 734.3 (7.4) | 12 268.3 (8.1) | 7 206.7 (7.9) |  |
| **Smoking status** |  |  |  |  |  | 0.042 |
| Never smoker, n (%) | 3 713.3 (51.5) | 24 263.1 (53.8) | 63 957.7 (54.5) | 82 693.9 (54.4) | 48 809.9 (53.8) |  |
| Previous smoker, n (%) | 2 578.8 (35.7) | 16 122.6 (35.8) | 41 235.4 (35.1) | 53 477.0 (35.2) | 31 698.2 (34.9) |  |
| Current smoker, n (%) | 924.7 (12.8) | 4 709.9 (10.4) | 12 148.7 (10.4) | 15 719.0 (10.3) | 10 190.3 (11.2) |  |
| **Acid inhibitor use, n (%)** | 262.4 (3.6) | 1 508.5 (3.3) | 3 555.0 (3.0) | 5 322.8 (3.5) | 3 055.3 (3.4) | 0.015 |
| **Recent hospital admissions, Mean (SD)** | 0.6 (1.2) | 0.5 (1.1) | 0.5 (1.1) | 0.5 (1.1) | 0.6 (1.2) | 0.033 |
| **Comorbidities** |  |  |  |  |  |  |
| Anxiety, n (%) | 301.8 (4.2) | 1 753.7 (3.9) | 4 422.3 (3.8) | 5 910.6 (3.9) | 3 518.5 (3.9) | 0.009 |
| Depression, n (%) | 723.2 (10.0) | 3 627.3 (8.0) | 9 497.7 (8.1) | 12 838.6 (8.5) | 7 494.9 (8.3) | 0.030 |
| Hypertension, n (%) | 2 103.1 (29.1) | 12 192.8 (27.0) | 31 412.9 (26.8) | 40 399.5 (26.6) | 24 794.2 (27.3) | 0.025 |
| Heart failure, n (%) | 32.1 (0.4) | 250.5 (0.6) | 716.5 (0.6) | 779.3 (0.5) | 521.4 (0.6) | 0.011 |
| Renal failure, n (%) | 60.1 (0.8) | 530.7 (1.2) | 1 562.0 (1.3) | 1 945.4 (1.3) | 1 083.2 (1.2) | 0.021 |
| Asthma, n (%) | 856.2 (11.9) | 5 742.3 (12.7) | 13 372.3 (11.4) | 17 939.9 (11.8) | 11 479.3 (12.7) | 0.022 |
| COPD, n (%) | 202.6 (2.8) | 816.5 (1.8) | 2 121.2 (1.8) | 2 817.9 (1.9) | 2 100.0 (2.3) | 0.034 |
| Diabetes, n (%) | 2 161.3 (29.9) | 12 568.6 (27.9) | 32 419.5 (27.6) | 41 692.7 (27.4) | 25 608.0 (28.2) | 0.025 |
| Thyroid disease, n (%) | 531.7 (7.4) | 3 088.5 (6.8) | 7 773.3 (6.6) | 10 322.4 (6.8) | 5 758.6 (6.3) | 0.018 |

SMD: standard mean difference; BMI: body mass index; MET: metabolic equivalent of task; COPD: chronic obstructive pulmonary disease; SD: standard deviation

**Supplementary Table 9. HRs (95% CIs) for digestive diseases by different healthy sleep score after using inverse probability weights**

| **Healthy sleep score** | **N** | **Case** | **HR (95% CI)** | **P value** |
| --- | --- | --- | --- | --- |
| Any digestive diseases |  |  |  |  |
| 0-1 | 6 906 | 2 503 | 1.00 (reference) | 1.00 |
| 2 | 34 135 | 10 883 | 0.87 (0.83-0.92) | <0.01 |
| 3 | 90 133 | 25 162 | 0.79 (0.75-0.82) | <0.01 |
| 4 | 122 248 | 29 864 | 0.72 (0.68-0.75) | <0.01 |
| 5 | 73 248 | 16 095 | 0.69 (0.66-0.73) | <0.01 |
| Dyspepsia |  |  |  |  |
| 0-1 | 9 661 | 316 | 1.00 (reference) | 1.00 |
| 2 | 45 208 | 1 198 | 0.82 (0.71-0.95) | <0.01 |
| 3 | 113 131 | 2 584 | 0.73 (0.64-0.84) | <0.01 |
| 4 | 148 098 | 2 953 | 0.66 (0.58-0.76) | <0.01 |
| 5 | 86 308 | 1 661 | 0.65 (0.56-0.74) | <0.01 |
| IBS |  |  |  |  |
| 0-1 | 9 171 | 361 | 1.00(reference) | 1.00 |
| 2 | 43 285 | 1 269 | 0.83 (0.72-0.96) | <0.01 |
| 3 | 109 331 | 2 439 | 0.67 (0.58-0.76) | <0.01 |
| 4 | 143 830 | 2 710 | 0.59 (0.51-0.67) | <0.01 |
| 5 | 83 944 | 1 454 | 0.53 (0.46-0.61) | <0.01 |
| Constipation |  |  |  |  |
| 0-1 | 9 824 | 856 | 1.00(reference) | 1.00 |
| 2 | 45 802 | 3 281 | 0.87 (0.79-0.95) | <0.01 |
| 3 | 114 443 | 6 892 | 0.79 (0.73-0.86) | <0.01 |
| 4 | 149 698 | 7 634 | 0.73 (0.67-0.79) | <0.01 |
| 5 | 87 081 | 4 012 | 0.71 (0.65-0.78) | <0.01 |
| Peptic ulcer |  |  |  |  |
| 0-1 | 9 551 | 325 | 1.00(reference) | 1.00 |
| 2 | 44 870 | 1 149 | 0.80 (0.69-0.92) | <0.01 |
| 3 | 112 245 | 2 321 | 0.71 (0.62-0.81) | <0.01 |
| 4 | 147 500 | 2 577 | 0.66 (0.58-0.75) | <0.01 |
| 5 | 86 145 | 1 248 | 0.65 (0.56-0.75) | <0.01 |
| GERD |  |  |  |  |
| 0-1 | 8 746 | 1 234 | 1.00(reference) | 1.00 |
| 2 | 41 654 | 5 230 | 0.91 (0.85-0.98) | 0.01 |
| 3 | 106 331 | 11 175 | 0.80 (0.75-0.86) | <0.01 |
| 4 | 140 975 | 12 136 | 0.70 (0.65-0.75) | <0.01 |
| 5 | 83 138 | 6 247 | 0.65 (0.61-0.70) | <0.01 |
| IBD |  |  |  |  |
| 0-1 | 9 852 | 85 | 1.00(reference) | 1.00 |
| 2 | 45 807 | 358 | 0.96 (0.73-1.26) | 0.77 |
| 3 | 114 195 | 801 | 0.90 (0.69-1.17) | 0.42 |
| 4 | 149 178 | 894 | 0.82 (0.64-1.07) | 0.14 |
| 5 | 86 865 | 479 | 0.87 (0.66-1.14) | 0.31 |
| Gallbladder disease |  |  |  |  |
| 0-1 | 9 438 | 624 | 1.00(reference) | 1.00 |
| 2 | 44 315 | 2 464 | 0.89 (0.80-0.98) | 0.02 |
| 3 | 111 194 | 5 081 | 0.82 (0.74-0.90) | <0.01 |
| 4 | 146 014 | 5 658 | 0.77 (0.69-0.84) | <0.01 |
| 5 | 85 120 | 3 020 | 0.77 (0.70-0.85) | <0.01 |
| Severe liver disease |  |  |  |  |
| 0-1 | 9 969 | 126 | 1.00 (reference) | 1.00 |
| 2 | 46 347 | 492 | 0.90 (0.72-1.14) | 0.38 |
| 3 | 115 441 | 891 | 0.77 (0.62-0.96) | 0.02 |
| 4 | 150 748 | 881 | 0.72 (0.58-0.89) | <0.01 |
| 5 | 87 699 | 368 | 0.72 (0.56-0.92) | <0.01 |
| NAFLD |  |  |  |  |
| 0-1 | 9 954 | 342 | 1.00 (reference) | 1.00 |
| 2 | 46 331 | 1 123 | 0.84 (0.73-0.96) | 0.01 |
| 3 | 115 426 | 1 932 | 0.69 (0.60-0.78) | <0.01 |
| 4 | 150 727 | 1 895 | 0.63 (0.55-0.72) | <0.01 |
| 5 | 87 658 | 825 | 0.58 (0.50-0.68) | <0.01 |
| Pancreatic disease |  |  |  |  |
| 0-1 | 9 927 | 153 | 1.00 (reference) | 1.00 |
| 2 | 46 160 | 653 | 1.03 (0.84-1.27) | 0.76 |
| 3 | 115 067 | 1 349 | 0.92 (0.75-1.12) | 0.42 |
| 4 | 150 319 | 1 471 | 0.86 (0.70-1.05) | 0.14 |
| 5 | 87 456 | 748 | 0.88 (0.71-1.09) | 0.23 |
| Diverticulosis |  |  |  |  |
| 0-1 | 9 468 | 1 508 | 1.00 (reference) | 1.00 |
| 2 | 44 416 | 6 361 | 0.90 (0.84-0.96) | <0.01 |
| 3 | 111 416 | 14 067 | 0.82 (0.77-0.87) | <0.01 |
| 4 | 146 397 | 16 128 | 0.76 (0.71-0.81) | <0.01 |
| 5 | 85 648 | 8 244 | 0.73 (0.68-0.78) | <0.01 |

IBS: irritable bowel syndrome; GERD: gastroesophageal reflux disease; IBD: inflammatory bowel disease; NAFLD: nonalcoholic fatty liver disease; HR: hazard ratio; CI: confidence interval. Weighted HRs after IPTW and 95% CIs are presented.

**Supplementary Table 10. HRs (95% CIs) for digestive diseases by different healthy sleep score in the sensitive analysis after excluding individuals who reported sleeping less than 4 hours or more than 11 hours**

| **Healthy sleep score** | **N** | **Case** | **HR (95% CI) *** | **P value** |
| --- | --- | --- | --- | --- |
| Any digestive diseases |  |  |  |  |
| 0-1 | 6 755 | 2 432 | 1.00(reference) | 1.00 |
| 2 | 33 759 | 10 728 | 0.94 (0.90-0.98) | <0.01 |
| 3 | 89 655 | 24 976 | 0.85 (0.82-0.89) | <0.01 |
| 4 | 122 083 | 29 810 | 0.78 (0.75-0.81) | <0.01 |
| 5 | 73 248 | 16 095 | 0.72 (0.69-0.76) | <0.01 |
| Dyspepsia |  |  |  |  |
| 0-1 | 9 395 | 304 | 1.00(reference) | 1.00 |
| 2 | 44 612 | 1 176 | 0.85 (0.75-0.97) | 0.01 |
| 3 | 112 442 | 2 556 | 0.77 (0.68-0.87) | <0.01 |
| 4 | 147 880 | 2 945 | 0.70 (0.62-0.79) | <0.01 |
| 5 | 86 308 | 1 661 | 0.66 (0.58-0.75) | <0.01 |
| IBS |  |  |  |  |
| 0-1 | 8 927 | 348 | 1.00(reference) | 1.00 |
| 2 | 42 733 | 1 243 | 0.80 (0.71-0.90) | <0.01 |
| 3 | 108 681 | 2 413 | 0.65 (0.58-0.73) | <0.01 |
| 4 | 143 615 | 2 699 | 0.57 (0.51-0.64) | <0.01 |
| 5 | 83 944 | 1 454 | 0.51 (0.45-0.57) | <0.01 |
| Constipation |  |  |  |  |
| 0-1 | 9 557 | 820 | 1.00(reference) | 1.00 |
| 2 | 45 196 | 3197 | 0.92 (0.85-0.99) | 0.04 |
| 3 | 113 761 | 6 805 | 0.84 (0.78-0.90) | <0.01 |
| 4 | 149 478 | 7 617 | 0.77 (0.72-0.83) | <0.01 |
| 5 | 87 081 | 4 012 | 0.73 (0.67-0.79) | <0.01 |
| Peptic ulcer |  |  |  |  |
| 0-1 | 9 297 | 320 | 1.00(reference) | 1.00 |
| 2 | 44 280 | 1 124 | 0.85 (0.75-0.96) | 0.01 |
| 3 | 111 578 | 2 292 | 0.76 (0.67-0.85) | <0.01 |
| 4 | 147 288 | 2 570 | 0.70 (0.63-0.79) | <0.01 |
| 5 | 86 145 | 1 248 | 0.64 (0.56-0.72) | <0.01 |
| GERD |  |  |  |  |
| 0-1 | 8 511 | 1 192 | 1.00(reference) | 1.00 |
| 2 | 41 130 | 5 141 | 0.98 (0.92-1.04) | 0.51 |
| 3 | 105 705 | 11 076 | 0.87 (0.82-0.93) | <0.01 |
| 4 | 140 771 | 12 116 | 0.76 (0.72-0.81) | <0.01 |
| 5 | 83 138 | 6 247 | 0.69 (0.64-0.73) | <0.01 |
| IBD |  |  |  |  |
| 0-1 | 9 583 | 82 | 1.00(reference) | 1.00 |
| 2 | 45 189 | 355 | 1.03 (0.81-1.31) | 0.83 |
| 3 | 113 501 | 794 | 0.99 (0.79-1.24) | 0.92 |
| 4 | 148 953 | 891 | 0.90 (0.72-1.14) | 0.39 |
| 5 | 86 865 | 479 | 0.89 (0.70-1.13) | 0.34 |
| Gallbladder disease |  |  |  |  |
| 0-1 | 9 187 | 596 | 1.00(reference) | 1.00 |
| 2 | 43 733 | 2 413 | 1.00 (0.91-1.10) | 0.98 |
| 3 | 110 525 | 5 046 | 0.93 (0.85-1.01) | 0.10 |
| 4 | 145 799 | 5 647 | 0.86 (0.79-0.94) | <0.01 |
| 5 | 85 120 | 3 020 | 0.83 (0.76-0.91) | <0.01 |
| Severe liver disease |  |  |  |  |
| 0-1 | 9 692 | 122 | 1.00(reference) | 1.00 |
| 2 | 45 725 | 475 | 1.08 (0.88-1.32) | 0.45 |
| 3 | 114 738 | 866 | 0.94 (0.77-1.13) | 0.50 |
| 4 | 150 525 | 877 | 0.84 (0.70-1.03) | 0.09 |
| 5 | 87 699 | 368 | 0.74 (0.60-0.92) | <0.01 |
| NAFLD |  |  |  |  |
| 0-1 | 9 679 | 324 | 1.00(reference) | 1.00 |
| 2 | 45 711 | 1 082 | 0.95 (0.84-1.08) | 0.42 |
| 3 | 114 723 | 1 908 | 0.82 (0.73-0.93) | <0.01 |
| 4 | 150 502 | 1 889 | 0.73 (0.64-0.82) | <0.01 |
| 5 | 87 658 | 825 | 0.63 (0.55-0.72) | <0.01 |
| Pancreatic disease |  |  |  |  |
| 0-1 | 9 654 | 144 | 1.00(reference) | 1.00 |
| 2 | 45 544 | 640 | 1.10 (0.92-1.32) | 0.29 |
| 3 | 114 367 | 1 334 | 1.02 (0.86-1.21) | 0.84 |
| 4 | 150 095 | 1 466 | 0.94 (0.79-1.12) | 0.47 |
| 5 | 87 456 | 748 | 0.89 (0.74-1.06) | 0.19 |
| Diverticulosis |  |  |  |  |
| 0-1 | 9 214 | 1 455 | 1.00(reference) | 1.00 |
| 2 | 43 832 | 6 259 | 0.97 (0.91-1.02) | 0.26 |
| 3 | 110 753 | 13 987 | 0.90 (0.85-0.95) | <0.01 |
| 4 | 146 179 | 16 103 | 0.83 (0.78-0.87) | <0.01 |
| 5 | 85 648 | 8 244 | 0.76 (0.72-0.81) | <0.01 |

IBS: irritable bowel syndrome; GERD: gastroesophageal reflux disease; IBD: inflammatory bowel disease; NAFLD: nonalcoholic fatty liver disease; HR: hazard ratio; CI: confidence interval.

*Adjusted for age and sex, ethnicity, body mass index, Townsend Deprivation Index, household income, education, acid inhibitor use, the number of hospital admissions three years before the baseline, smoking status, alcohol consumption, physical activity, hypertension, heart failure, MI, stroke, asthma, renal failure, COPD, thyroid disease, anxiety, depression, dementia, and diabetes.

**Supplementary Table 11.** **HRs (95% CIs) for digestive diseases by different healthy sleep score after excluding participants who were diagnosed with digestive diseases within 2 years after baseline**

| **Healthy sleep score** | **N** | **Case** | **HR (95% CI) *** | **P value** |
| --- | --- | --- | --- | --- |
| Any digestive diseases |  |  |  |  |
| 0-1 | 6 517 | 2 153 | 1.00(reference) | 1.00 |
| 2 | 32 571 | 9 474 | 0.94 (0.90-0.98) | <0.01 |
| 3 | 86 696 | 22 054 | 0.85 (0.82-0.89) | <0.01 |
| 4 | 118 309 | 26 312 | 0.78 (0.75-0.82) | <0.01 |
| 5 | 71 260 | 14 317 | 0.73 (0.70-0.77) | <0.01 |
| Dyspepsia |  |  |  |  |
| 0-1 | 9 513 | 247 | 1.00(reference) | 1.00 |
| 2 | 44 641 | 924 | 0.83 (0.72-0.96) | 0.01 |
| 3 | 112 084 | 2 091 | 0.79 (0.69-0.90) | <0.01 |
| 4 | 146 895 | 2 384 | 0.71 (0.62-0.81) | <0.01 |
| 5 | 85 664 | 1 333 | 0.67 (0.58-0.77) | <0.01 |
| IBS |  |  |  |  |
| 0-1 | 9 045 | 310 | 1.00(reference) | 1.00 |
| 2 | 42 787 | 1 055 | 0.78 (0.68-0.88) | <0.01 |
| 3 | 108 390 | 2 052 | 0.64 (0.57-0.72) | <0.01 |
| 4 | 142 747 | 2 265 | 0.56 (0.49-0.63) | <0.01 |
| 5 | 83 419 | 1 238 | 0.50 (0.44-0.57) | <0.01 |
| Constipation |  |  |  |  |
| 0-1 | 9 684 | 793 | 1.00(reference) | 1.00 |
| 2 | 45 283 | 3 036 | 0.92 (0.85-0.99) | 0.03 |
| 3 | 113 457 | 6 415 | 0.83 (0.77-0.90) | <0.01 |
| 4 | 148 623 | 7 144 | 0.77 (0.71-0.82) | <0.01 |
| 5 | 86 526 | 3 753 | 0.72 (0.67-0.78) | <0.01 |
| Peptic ulcer |  |  |  |  |
| 0-1 | 9 430 | 283 | 1.00(reference) | 1.00 |
| 2 | 44 481 | 1 037 | 0.90 (0.79-1.03) | 0.12 |
| 3 | 111 430 | 2 031 | 0.78 (0.68-0.88) | <0.01 |
| 4 | 146 558 | 2 249 | 0.71 (0.63-0.81) | <0.01 |
| 5 | 85 693 | 1 097 | 0.65 (0.57-0.75) | <0.01 |
| GERD |  |  |  |  |
| 0-1 | 8 547 | 1104 | 1.00(reference) | 1.00 |
| 2 | 40 860 | 4701 | 0.98 (0.92-1.05) | 0.53 |
| 3 | 104 756 | 10093 | 0.88 (0.82-0.93) | <0.01 |
| 4 | 139 186 | 10937 | 0.76 (0.71-0.80) | <0.01 |
| 5 | 82 250 | 5644 | 0.68 (0.64-0.73) | <0.01 |
| IBD |  |  |  |  |
| 0-1 | 9 759 | 74 | 1.00(reference) | 1.00 |
| 2 | 45 475 | 324 | 1.06 (0.82-1.37) | 0.65 |
| 3 | 113 523 | 693 | 0.99 (0.77-1.26) | 0.91 |
| 4 | 148 414 | 774 | 0.90 (0.71-1.15) | 0.41 |
| 5 | 86 498 | 425 | 0.91 (0.71-1.17) | 0.45 |
| Gallbladder disease |  |  |  |  |
| 0-1 | 9 277 | 539 | 1.00(reference) | 1.00 |
| 2 | 43 737 | 2 166 | 1.01 (0.92-1.11) | 0.89 |
| 3 | 110 029 | 4 447 | 0.93 (0.85-1.01) | 0.09 |
| 4 | 144 703 | 4 959 | 0.86 (0.79-0.94) | <0.01 |
| 5 | 84 445 | 2 647 | 0.83 (0.76-0.92) | <0.01 |
| Severe liver disease |  |  |  |  |
| 0-1 | 9 879 | 113 | 1.00(reference) | 1.00 |
| 2 | 46 022 | 453 | 1.14 (0.92-1.40) | 0.23 |
| 3 | 114 835 | 821 | 0.99 (0.81-1.21) | 0.91 |
| 4 | 150 055 | 818 | 0.88 (0.72-1.08) | 0.23 |
| 5 | 87 355 | 334 | 0.76 (0.61-0.95) | 0.01 |
| NAFLD |  |  |  |  |
| 0-1 | 9 855 | 325 | 1.00(reference) | 1.00 |
| 2 | 45 973 | 1 067 | 0.95 (0.84-1.08) | 0.46 |
| 3 | 114 762 | 1 837 | 0.81 (0.72-0.92) | <0.01 |
| 4 | 150 001 | 1 822 | 0.73 (0.64-0.82) | <0.01 |
| 5 | 87 309 | 798 | 0.63 (0.55-0.72) | <0.01 |
| Pancreatic disease |  |  |  |  |
| 0-1 | 9 837 | 141 | 1.00(reference) | 1.00 |
| 2 | 45 813 | 604 | 1.08 (0.90-1.30) | 0.39 |
| 3 | 114 409 | 1 247 | 1.00 (0.84-1.19) | 1.00 |
| 4 | 149 581 | 1 373 | 0.93 (0.78-1.11) | 0.41 |
| 5 | 87 084 | 692 | 0.87 (0.72-1.05) | 0.14 |
| Diverticulosis |  |  |  |  |
| 0-1 | 9 242 | 1 350 | 1.00(reference) | 1.00 |
| 2 | 43 476 | 5 696 | 0.96 (0.90-1.02) | 0.16 |
| 3 | 109 496 | 12 677 | 0.89 (0.84-0.94) | <0.01 |
| 4 | 144 295 | 14 620 | 0.82 (0.78-0.87) | <0.01 |
| 5 | 84 617 | 7 518 | 0.76 (0.72-0.81) | <0.01 |

IBS: irritable bowel syndrome; GERD: gastroesophageal reflux disease; IBD: inflammatory bowel disease; NAFLD: nonalcoholic fatty liver disease; HR: hazard ratio; CI: confidence interval.

*Adjusted for age and sex, ethnicity, body mass index, Townsend Deprivation Index, household income, education, acid inhibitor use, the number of hospital admissions three years before the baseline, smoking status, alcohol consumption, physical activity, hypertension, heart failure, MI, stroke, asthma, renal failure, COPD, thyroid disease, anxiety, depression, dementia, and diabetes.

**Supplementary Table 12. HRs (95% CIs) for digestive diseases in the sensitive analysis by different healthy sleep score after excluding individuals with missing covariates information**

| **Healthy sleep score** | **N** | **Case** | **HR (95% CI) *** | **P value** |
| --- | --- | --- | --- | --- |
| Any digestive diseases |  |  |  |  |
| 0-1 | 4 829 | 1 689 | 1.00(reference) | 1.00 |
| 2 | 24 404 | 7 508 | 0.94 (0.89-0.99) | 0.02 |
| 3 | 65 977 | 17 763 | 0.85 (0.81-0.90) | <0.01 |
| 4 | 91 386 | 21 557 | 0.78 (0.74-0.82) | <0.01 |
| 5 | 54 847 | 11 518 | 0.72 (0.68-0.76) | <0.01 |
| Dyspepsia |  |  |  |  |
| 0-1 | 6 658 | 213 | 1.00(reference) | 1.00 |
| 2 | 31 779 | 795 | 0.82 (0.71-0.96) | 0.01 |
| 3 | 81 796 | 1 774 | 0.75 (0.65-0.86) | <0.01 |
| 4 | 109 564 | 2 099 | 0.68 (0.59-0.78) | <0.01 |
| 5 | 64 154 | 1 184 | 0.64 (0.55-0.74) | <0.01 |
| IBS |  |  |  |  |
| 0-1 | 6 331 | 229 | 1.00(reference) | 1.00 |
| 2 | 30 492 | 851 | 0.83 (0.72-0.96) | 0.01 |
| 3 | 79 172 | 1 682 | 0.68 (0.59-0.78) | <0.01 |
| 4 | 106 455 | 1 915 | 0.59 (0.52-0.68) | <0.01 |
| 5 | 62 428 | 1 016 | 0.51 (0.44-0.60) | <0.01 |
| Constipation |  |  |  |  |
| 0-1 | 6 754 | 558 | 1.00(reference) | 1.00 |
| 2 | 32 173 | 2 113 | 0.88 (0.81-0.97) | <0.01 |
| 3 | 82 711 | 4666 | 0.83 (0.76-0.90) | <0.01 |
| 4 | 110 678 | 5254 | 0.75 (0.69-0.82) | <0.01 |
| 5 | 64 663 | 2810 | 0.72 (0.66-0.79) | <0.01 |
| Peptic ulcer |  |  |  |  |
| 0-1 | 6 574 | 228 | 1.00(reference) | 1.00 |
| 2 | 31 555 | 766 | 0.81 (0.70-0.94) | <0.01 |
| 3 | 81 145 | 1593 | 0.72 (0.63-0.83) | <0.01 |
| 4 | 109 049 | 1819 | 0.68 (0.59-0.78) | <0.01 |
| 5 | 64 023 | 863 | 0.60 (0.52-0.69) | <0.01 |
| GERD |  |  |  |  |
| 0-1 | 6 032 | 825 | 1.00(reference) | 1.00 |
| 2 | 29 400 | 3 535 | 0.96 (0.89-1.04) | 0.32 |
| 3 | 77 097 | 7 690 | 0.86 (0.80-0.92) | <0.01 |
| 4 | 104 589 | 8 586 | 0.74 (0.69-0.80) | <0.01 |
| 5 | 61 927 | 4 389 | 0.67 (0.62-0.72) | <0.01 |
| IBD |  |  |  |  |
| 0-1 | 6 764 | 56 | 1.00(reference) | 1.00 |
| 2 | 32 129 | 223 | 0.93 (0.69-1.25) | 0.63 |
| 3 | 82 464 | 538 | 0.94 (0.71-1.24) | 0.68 |
| 4 | 110 236 | 635 | 0.89 (0.68-1.18) | 0.41 |
| 5 | 64 504 | 344 | 0.88 (0.66-1.18) | 0.40 |
| Gallbladder disease |  |  |  |  |
| 0-1 | 6 521 | 407 | 1.00(reference) | 1.00 |
| 2 | 31 255 | 1 627 | 0.98 (0.87-1.09) | 0.65 |
| 3 | 80 515 | 3 441 | 0.91 (0.82-1.01) | 0.07 |
| 4 | 108 182 | 3 946 | 0.85 (0.76-0.94) | <0.01 |
| 5 | 63 324 | 2 102 | 0.81 (0.73-0.91) | <0.01 |
| Severe liver disease |  |  |  |  |
| 0-1 | 6 847 | 78 | 1.00(reference) | 1.00 |
| 2 | 32 513 | 356 | 1.27 (0.99-1.62) | 0.06 |
| 3 | 83 357 | 623 | 1.05 (0.83-1.33) | 0.70 |
| 4 | 111 378 | 622 | 0.92 (0.73-1.17) | 0.51 |
| 5 | 65 116 | 258 | 0.81 (0.62-1.05) | 0.10 |
| NAFLD |  |  |  |  |
| 0-1 | 6 836 | 223 | 1.00(reference) | 1.00 |
| 2 | 32 503 | 761 | 0.98 (0.84-1.13) | 0.76 |
| 3 | 83 337 | 1 284 | 0.79 (0.69-0.92) | <0.01 |
| 4 | 111 365 | 1 325 | 0.73 (0.63-0.84) | <0.01 |
| 5 | 65 082 | 572 | 0.63 (0.54-0.74) | <0.01 |
| Pancreatic disease |  |  |  |  |
| 0-1 | 6820 | 103 | 1.00(reference) | 1.00 |
| 2 | 32 385 | 441 | 1.05 (0.85-1.30) | 0.65 |
| 3 | 83 099 | 903 | 0.93 (0.76-1.15) | 0.51 |
| 4 | 111 091 | 1 022 | 0.87 (0.71-1.07) | 0.19 |
| 5 | 64 966 | 512 | 0.81 (0.65-1.01) | 0.06 |
| Diverticulosis |  |  |  |  |
| 0-1 | 6 514 | 1 019 | 1.00(reference) | 1.00 |
| 2 | 31 271 | 4 338 | 0.95 (0.89-1.02) | 0.16 |
| 3 | 80 640 | 9 876 | 0.88 (0.83-0.94) | <0.01 |
| 4 | 108 381 | 11 435 | 0.80 (0.75-0.86) | <0.01 |
| 5 | 63 676 | 5 787 | 0.73 (0.68-0.78) | <0.01 |

IBS: irritable bowel syndrome; GERD: gastroesophageal reflux disease; IBD: inflammatory bowel disease; NAFLD: nonalcoholic fatty liver disease; HR: hazard ratio; CI: confidence interval.

*Adjusted for age and sex, ethnicity, body mass index, Townsend Deprivation Index, household income, education, acid inhibitor use, the number of hospital admissions three years before the baseline, smoking status, alcohol consumption, physical activity, hypertension, heart failure, MI, stroke, asthma, renal failure, COPD, thyroid disease, anxiety, depression, dementia, and diabetes.

**Supplementary Table 13.** **HRs (95% CIs) for digestive diseases by different healthy sleep score in the sensitive analysis after limiting the censoring date to December 31, 2019**

| **Healthy sleep score** | **N** | **Case** | **HR (95% CI) *** | **P value** |
| --- | --- | --- | --- | --- |
| Any digestive diseases |  |  |  |  |
| 0-1 | 6 906 | 2 098 | 1.00(reference) | 1.00 |
| 2 | 34 135 | 8 964 | 0.93 (0.88-0.97) | <0.01 |
| 3 | 90 133 | 20 445 | 0.84 (0.80-0.87) | <0.01 |
| 4 | 122 248 | 24 028 | 0.76 (0.72-0.79) | <0.01 |
| 5 | 73 248 | 12 839 | 0.70 (0.67-0.73) | <0.01 |
| Dyspepsia |  |  |  |  |
| 0-1 | 9 661 | 295 | 1.00(reference) | 1.00 |
| 2 | 45 208 | 1 099 | 0.83 (0.73-0.95) | <0.01 |
| 3 | 113 131 | 2 422 | 0.77 (0.68-0.87) | <0.01 |
| 4 | 148 098 | 2 759 | 0.69 (0.61-0.78) | <0.01 |
| 5 | 86 308 | 1 560 | 0.66 (0.58-0.75) | <0.01 |
| IBS |  |  |  |  |
| 0-1 | 9 171 | 301 | 1.00(reference) | 1.00 |
| 2 | 43 285 | 1 068 | 0.81 (0.72-0.93) | <0.01 |
| 3 | 109 331 | 1 985 | 0.64 (0.57-0.73) | <0.01 |
| 4 | 143 830 | 2 278 | 0.58 (0.51-0.66) | <0.01 |
| 5 | 83 944 | 1 199 | 0.50 (0.44-0.57) | <0.01 |
| Constipation |  |  |  |  |
| 0-1 | 9 824 | 618 | 1.00(reference) | 1.00 |
| 2 | 45 802 | 2334 | 0.92 (0.84-1.00) | 0.06 |
| 3 | 114 443 | 4 768 | 0.82 (0.75-0.89) | <0.01 |
| 4 | 149 698 | 5 257 | 0.75 (0.69-0.81) | <0.01 |
| 5 | 87 081 | 2 759 | 0.70 (0.64-0.77) | <0.01 |
| Peptic ulcer |  |  |  |  |
| 0-1 | 9 551 | 271 | 1.00(reference) | 1.00 |
| 2 | 44 870 | 937 | 0.86 (0.75-0.99) | 0.03 |
| 3 | 112 245 | 1 903 | 0.78 (0.68-0.88) | <0.01 |
| 4 | 147 500 | 2 125 | 0.73 (0.64-0.83) | <0.01 |
| 5 | 86 145 | 997 | 0.64 (0.56-0.73) | <0.01 |
| GERD |  |  |  |  |
| 0-1 | 8 746 | 999 | 1.00(reference) | 1.00 |
| 2 | 41 654 | 4 175 | 0.97 (0.91-1.04) | 0.45 |
| 3 | 106 331 | 8 755 | 0.86 (0.80-0.92) | <0.01 |
| 4 | 140 975 | 9 480 | 0.75 (0.70-0.80) | <0.01 |
| 5 | 83 138 | 4 895 | 0.68 (0.63-0.73) | <0.01 |
| IBD |  |  |  |  |
| 0-1 | 9 852 | 72 | 1.00(reference) | 1.00 |
| 2 | 45 807 | 290 | 0.97 (0.75-1.26) | 0.83 |
| 3 | 114 195 | 617 | 0.89 (0.70-1.15) | 0.38 |
| 4 | 149 178 | 716 | 0.85 (0.67-1.09) | 0.20 |
| 5 | 86 865 | 396 | 0.86 (0.67-1.12) | 0.27 |
| Gallbladder disease |  |  |  |  |
| 0-1 | 9 438 | 505 | 1.00(reference) | 1.00 |
| 2 | 44 315 | 1 969 | 0.98 (0.89-1.08) | 0.73 |
| 3 | 111 194 | 3 993 | 0.90 (0.82-0.99) | 0.02 |
| 4 | 146 014 | 4 480 | 0.84 (0.77-0.92) | <0.01 |
| 5 | 85 120 | 2 372 | 0.80 (0.73-0.88) | <0.01 |
| Severe liver disease |  |  |  |  |
| 0-1 | 9 969 | 88 | 1.00(reference) | 1.00 |
| 2 | 46 347 | 369 | 1.20 (0.95-1.52) | 0.12 |
| 3 | 115 441 | 652 | 1.03 (0.82-1.29) | 0.81 |
| 4 | 150 748 | 614 | 0.88 (0.70-1.1) | 0.26 |
| 5 | 87 699 | 264 | 0.80 (0.62-1.02) | 0.07 |
| NAFLD |  |  |  |  |
| 0-1 | 9 954 | 233 | 1.00(reference) | 1.00 |
| 2 | 46 331 | 735 | 0.94 (0.81-1.09) | 0.38 |
| 3 | 115 426 | 1 215 | 0.78 (0.67-0.90) | <0.01 |
| 4 | 150 727 | 1 137 | 0.66 (0.57-0.76) | <0.01 |
| 5 | 87 658 | 490 | 0.57 (0.48-0.67) | <0.01 |
| Pancreatic disease |  |  |  |  |
| 0-1 | 9 927 | 112 | 1.00(reference) | 1.00 |
| 2 | 46 160 | 460 | 1.06 (0.86-1.30) | 0.61 |
| 3 | 115 067 | 957 | 0.99 (0.81-1.21) | 0.94 |
| 4 | 150 319 | 1 030 | 0.91 (0.74-1.11) | 0.33 |
| 5 | 87 456 | 507 | 0.83 (0.67-1.03) | 0.08 |
| Diverticulosis |  |  |  |  |
| 0-1 | 9 468 | 1 226 | 1.00(reference) | 1.00 |
| 2 | 4 4416 | 5 050 | 0.95 (0.89-1.01) | 0.08 |
| 3 | 111 416 | 11 007 | 0.87 (0.82-0.92) | <0.01 |
| 4 | 146 397 | 12 695 | 0.81 (0.76-0.86) | <0.01 |
| 5 | 85 648 | 6 392 | 0.73 (0.69-0.78) | <0.01 |

IBS: irritable bowel syndrome; GERD: gastroesophageal reflux disease; IBD: inflammatory bowel disease; NAFLD: nonalcoholic fatty liver disease; HR: hazard ratio; CI: confidence interval.

*Adjusted for age and sex, ethnicity, body mass index, Townsend Deprivation Index, household income, education, acid inhibitor use, the number of hospital admissions three years before the baseline, smoking status, alcohol consumption, physical activity, hypertension, heart failure, MI, stroke, asthma, renal failure, COPD, thyroid disease, anxiety, depression, dementia, and diabetes.

**Supplementary Table 14. HRs (95% CIs) for digestive diseases by different weighted score**

| **Weighted score** | **Case** | **Person-year** | **HR (95% CI) *** | **P value** |
| --- | --- | --- | --- | --- |
| Any digestive disease |  |  |  |  |
| 0~<=1 | 736 | 18 441.56 | 1.00(reference) | 1.00 |
| 1~<=2 | 8 051 | 267 185.93 | 0.90 (0.84-0.97) | 0.01 |
| 2~<=3 | 13 335 | 497 848.06 | 0.84 (0.78-0.91) | <0.01 |
| 3~<=4 | 21 311 | 926 011.37 | 0.78 (0.72-0.84) | <0.01 |
| 4~<=5 | 41 074 | 2 128 895.16 | 0.70 (0.65-0.75) | <0.01 |
| Dyspepsia |  |  |  |  |
| 0~<=1 | 125 | 44 175.40 | 1.00(reference) | 1.00 |
| 1~<=2 | 928 | 413 208.95 | 0.90 (0.75-1.09) | 0.27 |
| 2~<=3 | 1 543 | 745 215.84 | 0.85 (0.71-1.02) | 0.08 |
| 3~<=4 | 1 521 | 889 605.92 | 0.76 (0.63-0.91) | <0.01 |
| 4~<=5 | 4 595 | 3 194 114.23 | 0.68 (0.57-0.81) | <0.01 |
| IBS |  |  |  |  |
| 0~<=1 | 174 | 46 432.48 | 1.00(reference) | 1.00 |
| 1~<=2 | 302 | 109 521.43 | 0.82 (0.68-0.99) | 0.04 |
| 2~<=3 | 1 984 | 863 051.84 | 0.68 (0.59-0.80) | <0.01 |
| 3~<=4 | 1 398 | 773 735.33 | 0.60 (0.51-0.70) | <0.01 |
| 4~<=5 | 4 375 | 3 332 896.04 | 0.47 (0.41-0.55) | <0.01 |
| Constipation |  |  |  |  |
| 0~<=1 | 419 | 43 258.32 | 1.00(reference) | 1.00 |
| 1~<=2 | 329 | 44 212.91 | 0.85 (0.74-0.98) | 0.03 |
| 2~<=3 | 5 062 | 899 777.48 | 0.75 (0.68-0.83) | <0.01 |
| 3~<=4 | 5 749 | 1 246 833.91 | 0.67 (0.61-0.74) | <0.01 |
| 4~<=5 | 11 116 | 3 089 604.99 | 0.60 (0.54-0.66) | <0.01 |
| Peptic ulcer |  |  |  |  |
| 0~<=1 | 126 | 38 362.64 | 1.00(reference) | 1.00 |
| 1~<=2 | 1 438 | 710 381.89 | 0.86 (0.72-1.04) | 0.12 |
| 2~<=3 | 489 | 289 643.17 | 0.79 (0.65-0.97) | 0.02 |
| 3~<=4 | 2 201 | 1 401 230.69 | 0.74 (0.61-0.88) | <0.01 |
| 4~<=5 | 3 366 | 2 847 228.52 | 0.65 (0.54-0.77) | <0.01 |
| GERD |  |  |  |  |
| 0~<=1 | 1 171 | 101 990.87 | 1.00(reference) | 1.00 |
| 1~<=2 | 4 012 | 377 198.54 | 1.02 (0.95-1.08) | 0.64 |
| 2~<=3 | 6 601 | 706 360.91 | 0.95 (0.89-1.01) | 0.11 |
| 3~<=4 | 7 920 | 1 045 441.47 | 0.83 (0.78-0.88) | <0.01 |
| 4~<=5 | 16 318 | 2 628 188.32 | 0.73 (0.68-0.77) | <0.01 |
| IBD |  |  |  |  |
| 0~<=1 | 20 | 26 340.07 | 1.00(reference) | 1.00 |
| 1~<=2 | 454 | 720 556.12 | 1.04 (0.66-1.62) | 0.87 |
| 2~<=3 | 439 | 801 055.76 | 0.92 (0.59-1.44) | 0.71 |
| 3~<=4 | 1 185 | 2 612 468.79 | 0.84 (0.54-1.32) | 0.45 |
| 4~<=5 | 519 | 1 227 490.75 | 0.74 (0.47-1.16) | 0.19 |
| Gallbladder disease |  |  |  |  |
| 0~<=1 | 2 224 | 501 221.58 | 1.00(reference) | 1.00 |
| 1~<=2 | 3 103 | 836 623.81 | 0.90 (0.85-0.95) | <0.01 |
| 2~<=3 | 4 498 | 1 430 553.03 | 0.84 (0.80-0.88) | <0.01 |
| 3~<=4 | 6 851 | 2 358 198.04 | 0.80 (0.76-0.84) | <0.01 |
| 4~<=5 | 171 | 44 366.04 | 0.84 (0.72-0.98) | 0.03 |
| Severe liver disease |  |  |  |  |
| 0~<=1 | 415 | 564 637.77 | 1.00(reference) | 1.00 |
| 1~<=2 | 733 | 1 080 145.32 | 0.96 (0.85-1.09) | 0.56 |
| 2~<=3 | 375 | 647 657.49 | 0.84 (0.73-0.96) | 0.01 |
| 3~<=4 | 937 | 2 452 456.03 | 0.71 (0.63-0.79) | <0.01 |
| 4~<=5 | 298 | 708 745.60 | 0.64 (0.55-0.74) | <0.01 |
| NAFLD |  |  |  |  |
| 0~<=1 | 84 | 25 907.64 | 1.00(reference) | 1.00 |
| 1~<=2 | 591 | 276 531.37 | 0.93 (0.74-1.16) | 0.51 |
| 2~<=3 | 1 809 | 1 154 679.52 | 0.91 (0.73-1.13) | 0.40 |
| 3~<=4 | 1 963 | 1 780 383.30 | 0.78 (0.63-0.98) | 0.03 |
| 4~<=5 | 1 670 | 2 197 495.48 | 0.65 (0.52-0.81) | <0.01 |
| Pancreatic disease |  |  |  |  |
| 0~<=1 | 1 016 | 975 189.55 | 1.00(reference) | 1.00 |
| 1~<=2 | 466 | 487 090.56 | 0.96 (0.86-1.07) | 0.47 |
| 2~<=3 | 868 | 1 026 246.29 | 0.90 (0.82-0.98) | 0.02 |
| 3~<=4 | 1 971 | 2 873 775.07 | 0.83 (0.76-0.89) | <0.01 |
| 4~<=5 | 53 | 62 876.89 | 0.75 (0.57-0.98) | 0.04 |
| Diverticulosis |  |  |  |  |
| 0~<=1 | 1 359 | 102 089.31 | 1.00(reference) | 1.00 |
| 1~<=2 | 6 572 | 548 883.67 | 0.95 (0.90-1.01) | 0.09 |
| 2~<=3 | 11 926 | 1 177 715.20 | 0.88 (0.83-0.93) | <0.01 |
| 3~<=4 | 10 453 | 1 119 072.50 | 0.83 (0.78-0.88) | <0.01 |
| 4~<=5 | 15 998 | 2 071 935.38 | 0.76 (0.72-0.80) | <0.01 |

IBS: irritable bowel syndrome; GERD: gastroesophageal reflux disease; IBD: inflammatory bowel disease; NAFLD: nonalcoholic fatty liver disease; HR: hazard ratio; CI: confidence interval.

Weighted sleep score were constucted based on the 5 sleep factors by using the equation: weighted sleep score= (β1×factor1 +β2 ×factor 2 +…+β5×factor 5) × (5/sum of the β coefficients).

*Adjusted for age and sex, ethnicity, body mass index, Townsend Deprivation Index, household income, education, acid inhibitor use, the number of hospital admissions three years before the baseline, smoking status, alcohol consumption, physical activity, hypertension, heart failure, MI, stroke, asthma, renal failure, COPD, thyroid disease, anxiety, depression, dementia, and diabetes.

## Supplementary Table 15. Subgroups analysis of healthy sleep score with digestive diseases

| **Subgroup** | **Any digestive disease** | | **Dyspepsia** | | **IBS** | |
| --- | --- | --- | --- | --- | --- | --- |
|  | **HR (95% CI)** | **P interaction** | **HR (95% CI)** | **P interaction** | **HR (95% CI)** | **P interaction** |
| **Overall** | 0.92(0.91-0.92) |  | 0.91(0.89-0.93) |  | 0.85(0.84-0.87) |  |
| **Sex** |  | **0.0052** |  | 0.5982 |  | **0.0159** |
| Men | 0.92(0.91-0.93) |  | 0.92(0.88-0.95) |  | 0.87(0.84-0.91) |  |
| Women | 0.91(0.90-0.92) |  | 0.91(0.89-0.93) |  | 0.85(0.83-0.87) |  |
| **Age** |  | **<0.0001** |  | 0.9645 |  | **0.0027** |
| ≦60y | 0.91(0.90-0.92) |  | 0.92(0.89-0.95) |  | 0.84(0.82-0.86) |  |
| >60y | 0.93(0.92-0.94) |  | 0.91(0.88-0.93) |  | 0.88(0.85-0.91) |  |
| **Ethnicity** |  | 0.2213 |  | 0.1446 |  | 0.1114 |
| White | 0.92(0.91-0.93) |  | 0.91(0.89-0.93) |  | 0.86(0.84-0.88) |  |
| Other | 0.90(0.87-0.93) |  | 0.88(0.81-0.95) |  | 0.80(0.73-0.88) |  |
| **BMI, kg/m2** |  | **0.0016** |  | 0.0988 |  | 0.1639 |
| ≦mean(SD) | 0.91(0.90-0.92) |  | 0.93(0.90-0.95) |  | 0.87(0.84-0.89) |  |
| >mean(SD) | 0.92(0.91-0.93) |  | 0.88(0.85-0.92) |  | 0.84(0.80-0.87) |  |
| **Deprivation index** |  | 0.2872 |  | 0.4387 |  | 0.1077 |
| ≦mean(SD) | 0.91(0.91-0.92) |  | 0.91(0.88-0.93) |  | 0.87(0.84-0.89) |  |
| >mean(SD) | 0.92(0.91-0.93) |  | 0.91(0.88-0.94) |  | 0.84(0.81-0.86) |  |
| **Physical activity, MET minutes/week** |  | 0.5861 |  | 0.8627 |  | 0.2869 |
| ≦mean(SD) | 0.92(0.91-0.93) |  | 0.91(0.89-0.93) |  | 0.86(0.84-0.88) |  |
| >mean(SD) | 0.92(0.90-0.93) |  | 0.91(0.88-0.94) |  | 0.84(0.81-0.87) |  |
| **Household income, £** |  | **0.0003** |  | 0.3517 |  | 0.7998 |
| <18 000, n(%) | 0.92(0.91-0.93) |  | 0.88(0.85-0.92) |  | 0.85(0.82-0.89) |  |
| 18 000-30 999, n(%) | 0.93(0.92-0.94) |  | 0.93(0.90-0.97) |  | 0.85(0.82-0.89) |  |
| 31 000-51 999, n(%) | 0.91(0.90-0.92) |  | 0.91(0.88-0.95) |  | 0.84(0.81-0.88) |  |
| 52 000-100 000, n(%) | 0.91(0.89-0.92) |  | 0.94(0.89-0.99) |  | 0.87(0.83-0.92) |  |
| >100 000, n(%) | 0.91(0.88-0.94) |  | 0.90(0.80-1.01) |  | 0.87(0.78-0.97) |  |
| **Alcohol consumption** |  | 0.4225 |  | 0.9145 |  | **0.0009** |
| Daily or almost daily, n(%) | 0.93(0.91-0.94) |  | 0.94(0.89-0.99) |  | 0.90(0.85-0.94) |  |
| Three or four times a week, n(%) | 0.92(0.90-0.93) |  | 0.89(0.85-0.94) |  | 0.87(0.83-0.92) |  |
| Once or twice a week, n(%) | 0.92(0.91-0.94) |  | 0.91(0.87-0.95) |  | 0.86(0.82-0.89) |  |
| One to three times a month, n(%) | 0.91(0.89-0.93) |  | 0.90(0.85-0.96) |  | 0.85(0.80-0.91) |  |
| Special occasions only or never, n(%) | 0.92(0.90-0.94) |  | 0.93(0.89-0.99) |  | 0.82(0.78-0.86) |  |
| Never, n(%) | 0.89(0.87-0.91) |  | 0.88(0.83-0.94) |  | 0.80(0.75-0.85) |  |
| **Smoking status** |  | **0.0314** |  | 0.1091 |  | 0.4165 |
| Never smoker, n(%) | 0.91(0.90-0.92) |  | 0.90(0.88-0.93) |  | 0.85(0.82-0.87) |  |
| Previous smoker, n(%) | 0.92(0.91-0.93) |  | 0.92(0.89-0.95) |  | 0.86(0.83-0.89) |  |
| Current smoker, n(%) | 0.92(0.90-0.94) |  | 0.93(0.88-0.99) |  | 0.85(0.80-0.90) |  |
| **Acid inhibitor use** |  | **0.0245** |  | **0.0006** |  | **0.0037** |
| No | 0.92(0.91-0.92) |  | 0.91(0.89-0.93) |  | 0.85(0.83-0.87) |  |
| Yes | 0.94(0.90-0.98) |  | 1(0.92-1.08) |  | 0.94(0.86-1.03) |  |
| **Recent hospital admissions** |  | 0.4824 |  | 0.9368 |  | **0.0498** |
| ≦mean(SD) | 0.92(0.91-0.93) |  | 0.92(0.89-0.94) |  | 0.84(0.81-0.86) |  |
| >mean(SD) | 0.91(0.90-0.92) |  | 0.90(0.88-0.93) |  | 0.87(0.85-0.90) |  |
| **Education** |  | **0.0165** |  | 0.3888 |  | 0.7914 |
| No | 0.92(0.91-0.93) |  | 0.91(0.88-0.93) |  | 0.85(0.83-0.87) |  |
| Yes | 0.91(0.90-0.92) |  | 0.93(0.89-0.97) |  | 0.86(0.82-0.89) |  |
| **Comorbidities** |  |  |  |  |  |  |
| **Anxiety** |  | **0.0435** |  | 0.1543 |  | 0.0556 |
| No | 0.92(0.91-0.92) |  | 0.91(0.89-0.93) |  | 0.85(0.83-0.87) |  |
| Yes | 0.93(0.90-0.96) |  | 0.94(0.87-1.01) |  | 0.88(0.82-0.95) |  |
| **Depression** |  | 0.2596 |  | **0.0190** |  | 0.7170 |
| No | 0.92(0.91-0.92) |  | 0.90(0.88-0.92) |  | 0.85(0.83-0.87) |  |
| Yes | 0.92(0.90-0.94) |  | 0.96(0.90-1.01) |  | 0.86(0.81-0.90) |  |
| **Hypertension** |  | **<0.0001** |  | **0.0425** |  | 0.2165 |
| No | 0.91(0.90-0.92) |  | 0.93(0.90-0.95) |  | 0.85(0.83-0.87) |  |
| Yes | 0.93(0.92-0.94) |  | 0.88(0.85-0.91) |  | 0.86(0.83-0.90) |  |
| **Heart failure** |  | 0.2427 |  | 0.7894 |  | 0.6114 |
| No | 0.92(0.91-0.92) |  | 0.91(0.89-0.93) |  | 0.85(0.83-0.87) |  |
| Yes | 0.91(0.85-0.99) |  | 0.89(0.69-1.14) |  | 0.96(0.74-1.25) |  |
| **Renal failure** |  | 0.2554 |  | 0.2148 |  | 0.6652 |
| No | 0.92(0.91-0.92) |  | 0.91(0.89-0.93) |  | 0.85(0.84-0.87) |  |
| Yes | 0.91(0.86-0.96) |  | 0.82(0.71-0.95) |  | 0.80(0.69-0.93) |  |
| **Asthma** |  | **0.0150** |  | 0.9535 |  | 0.1997 |
| No | 0.92(0.91-0.92) |  | 0.91(0.89-0.93) |  | 0.86(0.84-0.88) |  |
| Yes | 0.93(0.91-0.95) |  | 0.90(0.86-0.95) |  | 0.82(0.78-0.87) |  |
| **COPD** |  | **0.0267** |  | 0.7954 |  | 0.1211 |
| No | 0.92(0.91-0.92) |  | 0.91(0.89-0.93) |  | 0.85(0.83-0.87) |  |
| Yes | 0.93(0.89-0.97) |  | 0.89(0.79-0.99) |  | 0.90(0.80-1.01) |  |
| **Diabetes** |  | **<0.0001** |  | **0.0219** |  | 0.2718 |
| No | 0.91(0.90-0.92) |  | 0.93(0.91-0.95) |  | 0.85(0.83-0.87) |  |
| Yes | 0.93(0.92-0.94) |  | 0.88(0.85-0.91) |  | 0.86(0.83-0.90) |  |
| **Thyroid disease** |  | **0.0249** |  | 0.6078 |  | **0.0010** |
| No | 0.92(0.91-0.92) |  | 0.91(0.89-0.93) |  | 0.85(0.83-0.86) |  |
| Yes | 0.93(0.91-0.96) |  | 0.90(0.84-0.97) |  | 0.93(0.87-0.99) |  |
| **Dementia** |  | 0.6343 |  | 0.0895 |  | 0.7047 |
| No | 0.92(0.91-0.92) |  | 0.91(0.89-0.93) |  | 0.85(0.84-0.87) |  |
| Yes | 1.10(0.72-1.68) |  | NA |  | NA |  |
| **Myocardial infarction** |  | 0.2523 |  | 0.6280 |  | 0.9828 |
| No | 0.92(0.91-0.92) |  | 0.91(0.89-0.93) |  | 0.85(0.84-0.87) |  |
| Yes | 0.91(0.88-0.95) |  | 0.88(0.79-0.99) |  | 0.81(0.71-0.93) |  |
| **Stroke** |  | 0.4707 |  | **0.0450** |  | 0.1439 |
| No | 0.92(0.91-0.92) |  | 0.91(0.90-0.93) |  | 0.85(0.83-0.87) |  |
| Yes | 0.91(0.87-0.95) |  | 0.77(0.66-0.89) |  | 0.95(0.81-1.12) |  |
| **Subgroup** | **Constipation** | | **Peptic ulcer** | | **GERD** | |
|  | **HR (95% CI)** | **P interaction** | **HR (95% CI)** | **P interaction** | **HR (95% CI)** | **P interaction** |
| **Overall** | 0.92(0.91-0.93) |  | 0.91(0.89-0.93) |  | 0.89(0.88-0.90) |  |
| **Sex** |  | 0.2452 |  | 0.2045 |  | **<0.0001** |
| Men | 0.93(0.91-0.95) |  | 0.92(0.89-0.95) |  | 0.91(0.89-0.92) |  |
| Women | 0.91(0.89-0.93) |  | 0.89(0.87-0.92) |  | 0.88(0.87-0.89) |  |
| **Age** |  | **0.0060** |  | **0.0254** |  | **<0.0001** |
| ≦60y | 0.91(0.89-0.93) |  | 0.90(0.87-0.93) |  | 0.88(0.87-0.89) |  |
| >60y | 0.93(0.91-0.94) |  | 0.92(0.89-0.95) |  | 0.91(0.90-0.92) |  |
| **Ethnicity** |  | 0.2922 |  | 0.8063 |  | 0.3108 |
| White | 0.92(0.91-0.93) |  | 0.91(0.89-0.93) |  | 0.89(0.88-0.90) |  |
| Other | 0.89(0.85-0.94) |  | 0.89(0.81-0.97) |  | 0.92(0.88-0.96) |  |
| **BMI, kg/m2** |  | 0.5030 |  | 0.7694 |  | **0.0008** |
| ≦mean(SD) | 0.92(0.91-0.94) |  | 0.91(0.88-0.93) |  | 0.88(0.87-0.89) |  |
| >mean(SD) | 0.91(0.89-0.93) |  | 0.90(0.87-0.94) |  | 0.90(0.89-0.92) |  |
| **Deprivation index** |  | 0.9960 |  | 0.8686 |  | 0.7801 |
| ≦mean(SD) | 0.92(0.90-0.93) |  | 0.91(0.88-0.94) |  | 0.90(0.88-0.91) |  |
| >mean(SD) | 0.92(0.90-0.94) |  | 0.90(0.88-0.93) |  | 0.89(0.87-0.90) |  |
| **Physical activity, MET minutes/week** |  | 0.2519 |  | 0.6464 |  | 0.1892 |
| ≦mean(SD) | 0.92(0.90-0.93) |  | 0.91(0.89-0.94) |  | 0.89(0.88-0.91) |  |
| >mean(SD) | 0.93(0.91-0.95) |  | 0.90(0.87-0.93) |  | 0.89(0.87-0.90) |  |
| **Household income, £** |  | 0.3819 |  | **0.0139** |  | **<0.0001** |
| <18 000, n(%) | 0.92(0.90-0.94) |  | 0.93(0.89-0.96) |  | 0.91(0.90-0.93) |  |
| 18 000-30 999, n(%) | 0.92(0.90-0.94) |  | 0.91(0.87-0.94) |  | 0.89(0.87-0.91) |  |
| 31 000-51 999, n(%) | 0.92(0.89-0.94) |  | 0.90(0.86-0.95) |  | 0.88(0.86-0.90) |  |
| 52 000-100 000, n(%) | 0.92(0.89-0.96) |  | 0.90(0.85-0.95) |  | 0.89(0.87-0.92) |  |
| >100 000, n(%) | 0.90(0.84-0.97) |  | 0.83(0.73-0.94) |  | 0.85(0.81-0.90) |  |
| **Alcohol consumption** |  | **0.0138** |  | 0.6366 |  | 0.9177 |
| Daily or almost daily, n(%) | 0.94(0.91-0.96) |  | 0.95(0.90-1.00) |  | 0.90(0.88-0.93) |  |
| Three or four times a week, n(%) | 0.94(0.91-0.97) |  | 0.90(0.85-0.94) |  | 0.90(0.88-0.92) |  |
| Once or twice a week, n(%) | 0.92(0.90-0.95) |  | 0.93(0.89-0.97) |  | 0.88(0.87-0.90) |  |
| One to three times a month, n(%) | 0.91(0.87-0.94) |  | 0.88(0.83-0.94) |  | 0.89(0.87-0.92) |  |
| Special occasions only or never, n(%) | 0.93(0.90-0.96) |  | 0.88(0.83-0.93) |  | 0.89(0.87-0.92) |  |
| Never, n(%) | 0.87(0.83-0.90) |  | 0.88(0.83-0.94) |  | 0.87(0.85-0.90) |  |
| **Smoking status** |  | 0.1068 |  | 0.3489 |  | 0.1377 |
| Never smoker, n(%) | 0.91(0.90-0.93) |  | 0.90(0.88-0.93) |  | 0.89(0.88-0.90) |  |
| Previous smoker, n(%) | 0.92(0.91-0.94) |  | 0.91(0.88-0.94) |  | 0.90(0.88-0.91) |  |
| Current smoker, n(%) | 0.94(0.90-0.97) |  | 0.92(0.87-0.97) |  | 0.88(0.85-0.91) |  |
| **Acid inhibitor use** |  | 0.2749 |  | 0.3422 |  | **0.0003** |
| No | 0.92(0.91-0.93) |  | 0.91(0.89-0.93) |  | 0.89(0.88-0.90) |  |
| Yes | 0.93(0.87-0.99) |  | 0.94(0.85-1.03) |  | 0.93(0.89-0.98) |  |
| **Recent hospital admissions** |  | 0.3851 |  | 0.6933 |  | **0.0018** |
| ≦mean(SD) | 0.92(0.91-0.94) |  | 0.91(0.88-0.94) |  | 0.88(0.87-0.90) |  |
| >mean(SD) | 0.91(0.90-0.93) |  | 0.91(0.88-0.94) |  | 0.90(0.89-0.92) |  |
| **Education** |  | 0.7948 |  | 0.6560 |  | **0.0041** |
| No | 0.92(0.91-0.93) |  | 0.91(0.88-0.93) |  | 0.90(0.89-0.91) |  |
| Yes | 0.92(0.90-0.95) |  | 0.92(0.88-0.96) |  | 0.88(0.86-0.90) |  |
| **Comorbidities** |  |  |  |  |  |  |
| **Anxiety** |  | 0.1392 |  | 0.2274 |  | **0.0017** |
| No | 0.92(0.91-0.94) |  | 0.91(0.89-0.93) |  | 0.89(0.88-0.90) |  |
| Yes | 0.87(0.82-0.91) |  | 0.85(0.78-0.93) |  | 0.93(0.89-0.97) |  |
| **Depression** |  | 0.1091 |  | 0.4633 |  | **0.0005** |
| No | 0.92(0.91-0.93) |  | 0.92(0.89-0.94) |  | 0.89(0.88-0.90) |  |
| Yes | 0.93(0.90-0.96) |  | 0.86(0.81-0.92) |  | 0.92(0.90-0.95) |  |
| **Hypertension** |  | 0.1615 |  | **0.0031** |  | **<0.0001** |
| No | 0.91(0.90-0.93) |  | 0.89(0.86-0.92) |  | 0.88(0.87-0.89) |  |
| Yes | 0.93(0.91-0.95) |  | 0.94(0.91-0.97) |  | 0.91(0.90-0.93) |  |
| **Heart failure** |  | 0.4574 |  | **0.0053** |  | **0.0164** |
| No | 0.92(0.91-0.93) |  | 0.91(0.89-0.93) |  | 0.89(0.88-0.90) |  |
| Yes | 0.88(0.79-0.99) |  | 1.13(0.92-1.40) |  | 0.99(0.87-1.11) |  |
| **Renal failure** |  | 0.6815 |  | 0.1719 |  | 0.1984 |
| No | 0.92(0.91-0.93) |  | 0.91(0.89-0.93) |  | 0.89(0.88-0.90) |  |
| Yes | 0.91(0.84-0.98) |  | 0.93(0.81-1.07) |  | 0.90(0.84-0.97) |  |
| **Asthma** |  | 0.4976 |  | 0.2837 |  | 0.1166 |
| No | 0.92(0.91-0.93) |  | 0.91(0.88-0.93) |  | 0.89(0.88-0.90) |  |
| Yes | 0.91(0.88-0.94) |  | 0.93(0.88-0.98) |  | 0.89(0.87-0.92) |  |
| **COPD** |  | **0.0297** |  | 0.4945 |  | **0.0035** |
| No | 0.92(0.91-0.93) |  | 0.91(0.89-0.93) |  | 0.89(0.88-0.90) |  |
| Yes | 0.95(0.90-1.01) |  | 0.91(0.81-1.01) |  | 0.93(0.88-0.98) |  |
| **Diabetes** |  | 0.1117 |  | **0.0006** |  | **<0.0001** |
| No | 0.91(0.90-0.93) |  | 0.89(0.86-0.91) |  | 0.88(0.87-0.89) |  |
| Yes | 0.93(0.91-0.95) |  | 0.94(0.91-0.97) |  | 0.91(0.90-0.93) |  |
| **Thyroid disease** |  | 0.0661 |  | 0.0628 |  | 0.0569 |
| No | 0.92(0.91-0.93) |  | 0.91(0.89-0.93) |  | 0.89(0.88-0.90) |  |
| Yes | 0.94(0.90-0.98) |  | 0.94(0.87-1.01) |  | 0.90(0.87-0.93) |  |
| **Dementia** |  | **0.0369** |  | 0.9522 |  | 0.4796 |
| No | 0.92(0.91-0.93) |  | 0.91(0.89-0.93) |  | 0.89(0.88-0.90) |  |
| Yes | 0.59(0.34-1.05) |  | NA |  | 0.23(0.14-0.38) |  |
| **Myocardial infarction** |  | 0.8869 |  | **0.0118** |  | **0.0003** |
| No | 0.92(0.91-0.93) |  | 0.90(0.88-0.92) |  | 0.89(0.88-0.90) |  |
| Yes | 0.90(0.85-0.96) |  | 0.98(0.89-1.07) |  | 0.95(0.90-1.00) |  |
| **Stroke** |  | 0.2041 |  | 0.1272 |  | 0.3071 |
| No | 0.92(0.91-0.93) |  | 0.91(0.89-0.93) |  | 0.89(0.88-0.90) |  |
| Yes | 0.95(0.88-1.01) |  | 0.96(0.84-1.08) |  | 0.91(0.84-0.97) |  |
| **Subgroup** | **IBD** | | **Gallbladder disease** | | **Severe liver disease** | |
|  | **HR (95% CI)** | **P interaction** | **HR (95% CI)** | **P interaction** | **HR (95% CI)** | **P interaction** |
| **Overall** | 0.95(0.92-0.99) |  | 0.94(0.93-0.96) |  | 0.90(0.87-0.94) |  |
| **Sex** |  | 0.3114 |  | 0.1513 |  | 0.8895 |
| Men | 0.96(0.91-1.01) |  | 0.95(0.92-0.97) |  | 0.91(0.87-0.95) |  |
| Women | 0.95(0.90-1.00) |  | 0.94(0.92-0.95) |  | 0.89(0.84-0.95) |  |
| **Age** |  | 0.9168 |  | **0.0020** |  | 0.9819 |
| ≦60y | 0.96(0.91-1.01) |  | 0.92(0.90-0.94) |  | 0.92(0.87-0.97) |  |
| >60y | 0.94(0.89-0.99) |  | 0.96(0.94-0.98) |  | 0.90(0.86-0.94) |  |
| **Ethnicity** |  | 0.1454 |  | 0.0544 |  | 0.4061 |
| White | 0.95(0.91-0.98) |  | 0.95(0.93-0.96) |  | 0.90(0.87-0.94) |  |
| Other | 1.04(0.89-1.21) |  | 0.88(0.82-0.95) |  | 0.94(0.79-1.13) |  |
| **BMI, kg/m2** |  | 0.0581 |  | **0.0039** |  | 0.8225 |
| ≦mean(SD) | 0.93(0.89-0.97) |  | 0.92(0.91-0.94) |  | 0.91(0.86-0.95) |  |
| >mean(SD) | 1.00(0.94-1.07) |  | 0.94(0.92-0.96) |  | 0.88(0.84-0.93) |  |
| **Deprivation index** |  | 0.9468 |  | 0.5546 |  | **0.0487** |
| ≦mean(SD) | 0.95(0.91-1.01) |  | 0.94(0.93-0.96) |  | 0.88(0.83-0.93) |  |
| >mean(SD) | 0.94(0.89-1.00) |  | 0.94(0.92-0.96) |  | 0.92(0.88-0.97) |  |
| **Physical activity, MET minutes/week** |  | 0.2978 |  | **0.0400** |  | 0.6916 |
| ≦mean(SD) | 0.97(0.92-1.01) |  | 0.93(0.91-0.94) |  | 0.90(0.86-0.94) |  |
| >mean(SD) | 0.93(0.87-0.99) |  | 0.98(0.95-1.00) |  | 0.92(0.86-0.98) |  |
| **Household income, £** |  | 0.5766 |  | **0.0001** |  | 0.8159 |
| <18 000, n(%) | 0.96(0.89-1.03) |  | 0.96(0.93-0.99) |  | 0.90(0.85-0.95) |  |
| 18 000-30 999, n(%) | 0.96(0.89-1.03) |  | 0.96(0.93-0.98) |  | 0.89(0.83-0.96) |  |
| 31 000-51 999, n(%) | 0.95(0.87-1.02) |  | 0.92(0.89-0.95) |  | 0.91(0.84-0.98) |  |
| 52 000-100 000, n(%) | 0.94(0.86-1.03) |  | 0.93(0.89-0.97) |  | 0.93(0.84-1.03) |  |
| >100 000, n(%) | 0.94(0.78-1.14) |  | 0.93(0.86-1.01) |  | 0.95(0.77-1.18) |  |
| **Alcohol consumption** |  | 0.1895 |  | 0.9929 |  | 0.1035 |
| Daily or almost daily, n(%) | 0.95(0.87-1.03) |  | 0.98(0.94-1.02) |  | 0.88(0.83-0.94) |  |
| Three or four times a week, n(%) | 0.98(0.90-1.07) |  | 0.96(0.93-1.00) |  | 0.97(0.89-1.06) |  |
| Once or twice a week, n(%) | 0.91(0.85-0.99) |  | 0.92(0.90-0.95) |  | 0.90(0.83-0.98) |  |
| One to three times a month, n(%) | 0.89(0.79-1.00) |  | 0.93(0.90-0.97) |  | 0.86(0.76-0.98) |  |
| Special occasions only or never, n(%) | 0.97(0.87-1.08) |  | 0.94(0.91-0.98) |  | 0.92(0.83-1.01) |  |
| Never, n(%) | 1.06(0.94-1.19) |  | 0.92(0.89-0.97) |  | 0.90(0.82-1.00) |  |
| **Smoking status** |  | 0.4605 |  | **0.0444** |  | 0.7134 |
| Never smoker, n(%) | 0.95(0.89-1.00) |  | 0.94(0.92-0.96) |  | 0.93(0.88-0.99) |  |
| Previous smoker, n(%) | 0.96(0.90-1.01) |  | 0.95(0.93-0.97) |  | 0.88(0.84-0.93) |  |
| Current smoker, n(%) | 0.96(0.87-1.06) |  | 0.94(0.90-0.98) |  | 0.90(0.83-0.98) |  |
| **Acid inhibitor use** |  | 0.3813 |  | **0.0296** |  | 0.1971 |
| No | 0.95(0.91-0.99) |  | 0.94(0.93-0.96) |  | 0.90(0.87-0.93) |  |
| Yes | 1.00(0.84-1.19) |  | 0.98(0.92-1.05) |  | 0.99(0.85-1.15) |  |
| **Recent hospital admissions** |  | 0.6778 |  | 0.2810 |  | 0.2569 |
| ≦mean(SD) | 0.97(0.92-1.02) |  | 0.94(0.93-0.96) |  | 0.89(0.85-0.94) |  |
| >mean(SD) | 0.93(0.88-0.99) |  | 0.94(0.92-0.96) |  | 0.91(0.87-0.96) |  |
| **Education** |  | 0.8477 |  | 0.2695 |  | 0.4027 |
| No | 0.95(0.91-0.99) |  | 0.94(0.93-0.96) |  | 0.90(0.86-0.93) |  |
| Yes | 0.96(0.89-1.04) |  | 0.95(0.92-0.98) |  | 0.94(0.87-1.01) |  |
| **Comorbidities** |  |  |  |  |  |  |
| **Anxiety** |  | 0.7414 |  | 0.4640 |  | 0.9021 |
| No | 0.95(0.91-0.99) |  | 0.94(0.93-0.96) |  | 0.91(0.87-0.94) |  |
| Yes | 0.98(0.82-1.17) |  | 0.93(0.87-0.99) |  | 0.89(0.77-1.02) |  |
| **Depression** |  | 0.9994 |  | 0.1849 |  | 0.5461 |
| No | 0.95(0.92-0.99) |  | 0.94(0.93-0.96) |  | 0.91(0.87-0.94) |  |
| Yes | 0.93(0.83-1.05) |  | 0.94(0.91-0.98) |  | 0.91(0.83-1.00) |  |
| **Hypertension** |  | 0.4209 |  | **0.0059** |  | 0.3478 |
| No | 0.95(0.91-0.99) |  | 0.94(0.93-0.96) |  | 0.90(0.85-0.94) |  |
| Yes | 0.96(0.90-1.02) |  | 0.95(0.92-0.97) |  | 0.92(0.87-0.96) |  |
| **Heart failure** |  | 0.4994 |  | 0.1899 |  | 0.5477 |
| No | 0.95(0.92-0.99) |  | 0.94(0.93-0.96) |  | 0.90(0.87-0.94) |  |
| Yes | 1.08(0.77-1.53) |  | 0.99(0.85-1.16) |  | 0.91(0.73-1.13) |  |
| **Renal failure** |  | 0.4882 |  | 0.4059 |  | 0.7740 |
| No | 0.96(0.92-0.99) |  | 0.94(0.93-0.96) |  | 0.91(0.87-0.94) |  |
| Yes | 0.85(0.66-1.09) |  | 0.94(0.85-1.04) |  | 0.85(0.71-1.02) |  |
| **Asthma** |  | 0.6963 |  | 0.4004 |  | 0.3839 |
| No | 0.96(0.92-1.00) |  | 0.94(0.93-0.96) |  | 0.90(0.86-0.93) |  |
| Yes | 0.92(0.83-1.01) |  | 0.94(0.90-0.97) |  | 0.95(0.87-1.04) |  |
| **COPD** |  | 0.2062 |  | **0.0219** |  | **0.0051** |
| No | 0.95(0.91-0.99) |  | 0.94(0.93-0.96) |  | 0.89(0.86-0.93) |  |
| Yes | 1.01(0.83-1.23) |  | 0.98(0.90-1.05) |  | 1.07(0.92-1.24) |  |
| **Diabetes** |  | 0.3692 |  | **0.0044** |  | 0.1555 |
| No | 0.95(0.91-1.00) |  | 0.94(0.93-0.96) |  | 0.89(0.84-0.94) |  |
| Yes | 0.96(0.90-1.02) |  | 0.95(0.92-0.97) |  | 0.92(0.88-0.97) |  |
| **Thyroid disease** |  | 0.8631 |  | 0.1439 |  | 0.5180 |
| No | 0.95(0.92-0.99) |  | 0.94(0.93-0.96) |  | 0.90(0.87-0.94) |  |
| Yes | 0.95(0.83-1.08) |  | 0.96(0.92-1.01) |  | 0.92(0.80-1.04) |  |
| **Dementia** |  | 0.9211 |  | 0.7305 |  | 0.6672 |
| No | 0.95(0.92-0.99) |  | 0.94(0.93-0.96) |  | 0.90(0.87-0.94) |  |
| Yes | NA |  | NA |  | NA |  |
| **Myocardial infarction** |  | 0.0717 |  | 0.1564 |  | 0.3126 |
| No | 0.95(0.91-0.98) |  | 0.94(0.93-0.96) |  | 0.90(0.87-0.94) |  |
| Yes | 1.07(0.90-1.27) |  | 0.92(0.86-0.99) |  | 0.92(0.81-1.05) |  |
| **Stroke** |  | 0.7078 |  | 0.1624 |  | 0.2905 |
| No | 0.96(0.92-0.99) |  | 0.94(0.93-0.96) |  | 0.90(0.87-0.94) |  |
| Yes | 0.85(0.68-1.07) |  | 0.98(0.89-1.08) |  | 0.97(0.80-1.16) |  |
| **Subgroup** | **NAFLD** | | **Pancreatic disease** | | **Diverticulosis** | |
|  | **HR (95% CI)** | **P interaction** | **HR (95% CI)** | **P interaction** | **HR (95% CI)** | **P interaction** |
| **Overall** | 0.88(0.86-0.90) |  | 0.94(0.91-0.97) |  | 0.93(0.92-0.93) |  |
| **Sex** |  | 0.2989 |  | 0.4484 |  | 0.6935 |
| Men | 0.89(0.86-0.93) |  | 0.93(0.89-0.97) |  | 0.92(0.91-0.94) |  |
| Women | 0.87(0.84-0.90) |  | 0.94(0.91-0.98) |  | 0.93(0.92-0.94) |  |
| **Age** |  | 0.2654 |  | 0.2427 |  | **<0.0001** |
| ≦60y | 0.87(0.84-0.90) |  | 0.95(0.91-0.99) |  | 0.91(0.90-0.92) |  |
| >60y | 0.89(0.86-0.93) |  | 0.94(0.90-0.97) |  | 0.94(0.93-0.95) |  |
| **Ethnicity** |  | 0.0621 |  | 0.6595 |  | 0.4865 |
| White | 0.89(0.87-0.91) |  | 0.94(0.91-0.97) |  | 0.93(0.92-0.93) |  |
| Other | 0.79(0.72-0.86) |  | 0.96(0.83-1.10) |  | 0.91(0.87-0.96) |  |
| **BMI, kg/m2** |  | **0.0009** |  | **0.0172** |  | **<0.0001** |
| ≦mean(SD) | 0.85(0.82-0.88) |  | 0.92(0.88-0.95) |  | 0.91(0.90-0.92) |  |
| >mean(SD) | 0.88(0.85-0.91) |  | 0.96(0.92-1.01) |  | 0.95(0.93-0.96) |  |
| **Deprivation index** |  | 0.2014 |  | 0.8083 |  | 0.6833 |
| ≦mean(SD) | 0.88(0.85-0.91) |  | 0.94(0.90-0.98) |  | 0.93(0.92-0.94) |  |
| >mean(SD) | 0.88(0.85-0.91) |  | 0.94(0.90-0.98) |  | 0.93(0.91-0.94) |  |
| **Physical activity, MET minutes/week** |  | 0.5105 |  | 0.3220 |  | 0.3710 |
| ≦mean(SD) | 0.88(0.85-0.90) |  | 0.95(0.91-0.98) |  | 0.92(0.91-0.93) |  |
| >mean(SD) | 0.89(0.86-0.93) |  | 0.92(0.88-0.97) |  | 0.93(0.92-0.95) |  |
| **Household income, £** |  | 0.3840 |  | 0.4251 |  | **<0.0001** |
| <18 000, n(%) | 0.88(0.85-0.92) |  | 0.92(0.88-0.97) |  | 0.94(0.92-0.95) |  |
| 18 000-30 999, n(%) | 0.86(0.82-0.90) |  | 0.96(0.91-1.01) |  | 0.94(0.93-0.96) |  |
| 31 000-51 999, n(%) | 0.89(0.85-0.94) |  | 0.95(0.89-1.01) |  | 0.91(0.89-0.92) |  |
| 52 000-100 000, n(%) | 0.89(0.83-0.94) |  | 0.93(0.86-1.01) |  | 0.92(0.90-0.94) |  |
| >100 000, n(%) | 0.97(0.85-1.10) |  | 0.90(0.77-1.06) |  | 0.91(0.87-0.95) |  |
| **Alcohol consumption** |  | **0.0073** |  | **0.0067** |  | **0.0125** |
| Daily or almost daily, n(%) | 0.85(0.80-0.89) |  | 0.90(0.85-0.97) |  | 0.93(0.91-0.94) |  |
| Three or four times a week, n(%) | 0.95(0.89-1.01) |  | 0.97(0.91-1.04) |  | 0.92(0.91-0.94) |  |
| Once or twice a week, n(%) | 0.88(0.84-0.93) |  | 0.91(0.86-0.96) |  | 0.91(0.90-0.93) |  |
| One to three times a month, n(%) | 0.86(0.80-0.92) |  | 0.93(0.86-1.01) |  | 0.93(0.91-0.96) |  |
| Special occasions only or never, n(%) | 0.88(0.83-0.93) |  | 0.94(0.87-1.01) |  | 0.95(0.92-0.97) |  |
| Never, n(%) | 0.88(0.82-0.93) |  | 1.02(0.93-1.11) |  | 0.93(0.90-0.96) |  |
| **Smoking status** |  | 0.0744 |  | 0.5616 |  | 0.0866 |
| Never smoker, n(%) | 0.88(0.85-0.91) |  | 0.94(0.90-0.98) |  | 0.92(0.91-0.94) |  |
| Previous smoker, n(%) | 0.88(0.84-0.91) |  | 0.93(0.89-0.97) |  | 0.93(0.91-0.94) |  |
| Current smoker, n(%) | 0.89(0.84-0.95) |  | 0.97(0.90-1.04) |  | 0.93(0.90-0.95) |  |
| **Acid inhibitor use** |  | 0.0565 |  | 0.0637 |  | 0.5046 |
| No | 0.88(0.86-0.90) |  | 0.93(0.91-0.96) |  | 0.93(0.92-0.93) |  |
| Yes | 0.93(0.84-1.02) |  | 1.06(0.93-1.21) |  | 0.92(0.89-0.96) |  |
| **Recent hospital admissions** |  | 0.6242 |  | 0.7679 |  | 0.0764 |
| ≦mean(SD) | 0.89(0.86-0.92) |  | 0.93(0.90-0.97) |  | 0.92(0.91-0.94) |  |
| >mean(SD) | 0.87(0.84-0.90) |  | 0.94(0.90-0.98) |  | 0.93(0.91-0.94) |  |
| **Education** |  | 0.5580 |  | 0.2571 |  | **0.0159** |
| No | 0.88(0.85-0.90) |  | 0.94(0.91-0.98) |  | 0.93(0.92-0.94) |  |
| Yes | 0.90(0.85-0.94) |  | 0.93(0.87-0.99) |  | 0.92(0.90-0.93) |  |
| **Comorbidities** |  |  |  |  |  |  |
| **Anxiety** |  | **0.0181** |  | 0.5603 |  | 0.2981 |
| No | 0.88(0.85-0.90) |  | 0.94(0.91-0.96) |  | 0.93(0.92-0.93) |  |
| Yes | 0.96(0.87-1.05) |  | 1.00(0.88-1.14) |  | 0.93(0.89-0.96) |  |
| **Depression** |  | 0.3646 |  | **0.0381** |  | 0.1463 |
| No | 0.88(0.86-0.91) |  | 0.93(0.90-0.96) |  | 0.93(0.92-0.93) |  |
| Yes | 0.87(0.82-0.92) |  | 1.02(0.94-1.11) |  | 0.93(0.91-0.96) |  |
| **Hypertension** |  | **0.0118** |  | 0.9821 |  | **0.0001** |
| No | 0.88(0.85-0.91) |  | 0.95(0.91-0.98) |  | 0.92(0.91-0.93) |  |
| Yes | 0.88(0.85-0.92) |  | 0.93(0.89-0.98) |  | 0.94(0.92-0.95) |  |
| **Heart failure** |  | 0.1383 |  | 0.5420 |  | 0.0664 |
| No | 0.88(0.86-0.90) |  | 0.94(0.91-0.97) |  | 0.93(0.92-0.93) |  |
| Yes | 0.91(0.74-1.12) |  | 0.89(0.69-1.15) |  | 0.97(0.88-1.07) |  |
| **Renal failure** |  | 0.6555 |  | 0.2588 |  | 0.9222 |
| No | 0.88(0.86-0.90) |  | 0.94(0.91-0.97) |  | 0.93(0.92-0.93) |  |
| Yes | 0.87(0.76-1.00) |  | 0.94(0.79-1.10) |  | 0.91(0.85-0.97) |  |
| **Asthma** |  | **0.0110** |  | 0.3461 |  | **0.0039** |
| No | 0.88(0.85-0.90) |  | 0.94(0.91-0.97) |  | 0.92(0.91-0.93) |  |
| Yes | 0.90(0.85-0.95) |  | 0.95(0.88-1.02) |  | 0.94(0.92-0.96) |  |
| **COPD** |  | **0.0180** |  | 0.0999 |  | **0.0211** |
| No | 0.88(0.86-0.90) |  | 0.94(0.91-0.96) |  | 0.93(0.92-0.93) |  |
| Yes | 0.93(0.83-1.04) |  | 1.00(0.87-1.15) |  | 0.95(0.90-1.00) |  |
| **Diabetes** |  | **0.0300** |  | 0.9695 |  | **<0.0001** |
| No | 0.89(0.86-0.92) |  | 0.95(0.91-0.99) |  | 0.92(0.91-0.93) |  |
| Yes | 0.88(0.85-0.91) |  | 0.93(0.89-0.97) |  | 0.94(0.92-0.95) |  |
| **Thyroid disease** |  | **0.0290** |  | 0.6973 |  | **0.0068** |
| No | 0.88(0.85-0.90) |  | 0.94(0.91-0.97) |  | 0.92(0.91-0.93) |  |
| Yes | 0.92(0.86-1.00) |  | 0.94(0.85-1.03) |  | 0.96(0.93-0.99) |  |
| **Dementia** |  | 0.2634 |  | 0.7574 |  | 0.2771 |
| No | 0.88(0.86-0.90) |  | 0.94(0.91-0.97) |  | 0.93(0.92-0.93) |  |
| Yes | NA |  | NA |  | 1.24(0.82-1.85) |  |
| **Myocardial infarction** |  | 0.5891 |  | 0.6007 |  | **0.0192** |
| No | 0.89(0.86-0.91) |  | 0.94(0.92-0.97) |  | 0.92(0.92-0.93) |  |
| Yes | 0.80(0.72-0.89) |  | 0.87(0.77-0.99) |  | 0.96(0.91-1.00) |  |
| **Stroke** |  | **0.0394** |  | 0.2118 |  | 0.9943 |
| No | 0.88(0.86-0.90) |  | 0.94(0.91-0.97) |  | 0.93(0.92-0.93) |  |
| Yes | 0.89(0.78-1.02) |  | 1.00(0.84-1.20) |  | 0.90(0.84-0.95) |  |

GERD: gastroesophageal reflux disease; NAFLD: nonalcoholic fatty liver disease; HR: hazard ratio; CI: confidence interval; BMI: body mass index; MET: metabolic equivalent of task; COPD: chronic obstructive pulmonary disease; SD: standard deviation.

Adjusted HRs and 95% CIs are presented.
